# Plasma proteomic analysis of stable coronary artery disease indicates impairment of reverse cholesterol pathway

**DOI:** 10.1038/srep28042

**Published:** 2016-06-28

**Authors:** Trayambak Basak, Vinay Singh Tanwar, Gourav Bhardwaj, Nitin Bhardwaj, Shadab Ahmad, Gaurav Garg, Sreenivas V, Ganesan Karthikeyan, Sandeep Seth, Shantanu Sengupta

**Affiliations:** 1 Genomics and Molecular Medicine Unit, CSIR-Institute of Genomics and Integrative Biology, New Delhi, India; 2 Academy of Scientific & Innovative Research, New Delhi, India; 3 Department of Biostatistics, All India Institute of Medical Sciences, New Delhi, India; 4 Department of Cardiology, All India Institute of Medical Sciences, New Delhi, India.

## Abstract

Coronary artery disease (CAD) is one of the largest causes of death worldwide yet the traditional risk factors, although useful in identifying people at high risk, lack the desired predictive accuracy. Techniques like quantitative plasma proteomics holds immense potential to identify newer markers and this study (conducted in three phases) was aimed to identify differentially expressed proteins in stable CAD patients. In the first (discovery) phase, plasma from CAD cases (angiographically proven) and controls were subjected to iTRAQ based proteomic analysis. Proteins found to be differentially expressed were then validated in the second and third (verification and validation) phases in larger number of (n = 546) samples. After multivariate logistic regression adjusting for confounding factors (age, diet, etc.), four proteins involved in the reverse cholesterol pathway (Apo A1, ApoA4, Apo C1 and albumin) along with diabetes and hypertension were found to be significantly associated with CAD and could account for approximately 88% of the cases as revealed by ROC analysis. The maximum odds ratio was found to be 6.70 for albumin (p < 0.0001), followed by Apo AI (5.07, p < 0.0001), Apo CI (4.03, p = 0.001), and Apo AIV (2.63, p = 0.003). Down-regulation of apolipoproteins and albumin implicates the impairment of reverse cholesterol pathway in CAD.

Coronary artery disease (CAD) has remained as one of the most important causes of mortality and morbidity worldwide. According to WHO, almost seven million deaths occur yearly due to this disease^1^. It is estimated that global cardiovascular death would increase from 17.1 million (in 2004) to 23.4 million by 2030 with CAD contributing a significant proportion^2^. Furthermore, the burden of this disease has increased rapidly in the developing countries^3^. In India alone the mortality due to CAD has increased from 1.17 million to 1.59 million from 1990 to early 2000^4^. Since CAD is a complex disorder where both genetic and lifestyle (including dietary habits) contribute significantly, finding new potential markers holds its own clinical importance specifically in early detection and efficient management of the disease^5^.

In recent years, several studies focussed to identify genetic markers that could be associated with CAD. To this end several Genome Wide Association Studies (GWAS) have been undertaken by various groups to identify single nucleotide polymorphisms (SNPs) that are associated with CAD^6^,^7^,^8^,^9^. However, other than a few SNPs most of them could not be replicated in different populations. Further, most of the SNPs have low discriminative accuracy and the common variants account for about 10% of predicted genetic heritability of CAD^5^. Thus, even now, the classical risk factors in blood like total cholesterol, HDL, LDL etc. are routinely determined to assess the risk of CAD^1^. These traditional risk factors are useful in identifying people at high risk of developing CAD. Wang *et al*. showed a predictive accuracy of 0.7 area under the curve with classical risk factors for major cardiovascular events in Framingham heart study. However, identification of newer markers are necessary to increase the predictive accuracy especially since various prospective studies using the classical markers of CAD did not show a high predictive significance for the disease^10^,^11^.

With the advent of mass spectrometry based high throughput proteomic technologies, protein markers have gained attention as it is feasible to compare the proteome of diseased and healthy individuals and identify differentially expressed proteins that could potentially act as disease markers. Proteomics has helped in identifying markers for several diseases like cancer, neurological diseases etc^12^,^13^,^14^. Although several groups have also attempted to identify markers for various cardiovascular diseases including acute coronary syndrome, unstable and stable angina, myocardial infarction etc^15^,^16^,^17^, surprisingly, studies on identifying markers for stable coronary artery disease are limited. A couple of studies have been done based on peptide profiling in urine and a few peptide signatures were identified as potential biomarkers^18^,^19^. However the use of peptide signatures has an inherent problem since they can be detected only using mass spectrometer thereby limiting their utility as biomarkers in clinical practice as mass spectrometers are till date not routinely used as a tool for biomarker profiling^20^,^21^. Donahue *et al*. reported a qualitative proteomic analysis using pooled plasma from 53 CAD cases and 53 controls^22^. However, qualitative proteomic measurement will have limited application in terms of biomarkers. Thus, a panel of defined quantifiable proteins (and not peptide signatures) from an easily accessible biological fluid (like urine or plasma) is necessary for it to be considered as potential biomarkers^23^.

In the present study using high throughput iTRAQ (Isobaric tag for relative and absolute quantitation) based relative quantitation of plasma proteome we have identified proteins that are differentially expressed in stable CAD patients. The differentially expressed proteins in the discovery phase were further validated in two phases in more than 500 samples. After multivariate logistic regression we report four proteins involved in the reverse cholesterol pathway to be significantly associated with CAD.

## Results

**Figure 1:**
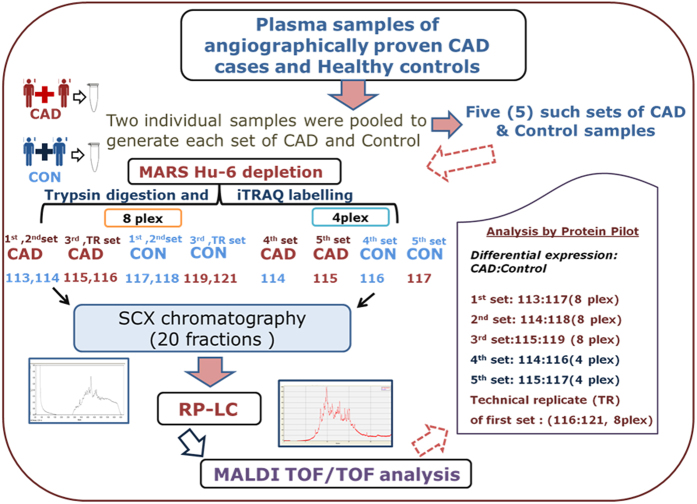
Schematic workflow for the iTRAQ based discovery phase analysis.

In this study we attempted to identify proteins that are differentially expressed in stable CAD patients. The study was conducted in three phases. In the discovery (1^st^) phase a total of 20 plasma samples (10 CAD cases and 10 controls) were selected for iTRAQ experiments. In the verification (2^nd^) phase, samples from 40 individuals (20 CAD cases and 20 controls) were assayed for proteins that were found to be differentially expressed in the discovery phase either using ELISA or Biochemical analyser. Proteins whose concentration in CAD samples remained significantly different in CAD patients in the verification phase were finally validated (3^rd^ phase) in 506 samples (253 CAD cases and 253 controls) (Fig. 1). The demographic characteristics of the samples used in the three phases are shown in Table 1.

### Discovery Phase

In the discovery phase 10 CAD cases and 10 controls were subjected to differential proteomic analysis after pooling two samples of same age and sex to minimize the intra-individual variation. The top six abundant proteins in the pooled plasma were then depleted using Multiple affinity removal system (MARS, Hu-6) as mentioned in the methods section. These depleted case- control samples were then subjected to two different sets of iTRAQ experiments. Since, both diabetes and hypertension are risk factors for CAD we compared cases with or without diabetes and/or hypertension with controls having similar status in terms of these two risk factors, thus, minimizing the effects of the two confounding risk factors of CAD. For the first iTRAQ experiment (8 plex) all the patients selected were suffering from triple vessel disease while in the second experiment (4 plex) double vessel stable CAD patients were considered.

A total of 214 and 220 proteins at 1% FDR (unused score ≥2.0) were identified in the 8 plex and 4 plex iTRAQ experiments respectively (Supplementary Table 1). For quantitation purposes, those proteins that were identified with two or more unique peptides in at least one experiment with an unused score ≥2.0 were only considered. There were 122 and 159 such high confidence identified proteins in the two experiments respectively with 111 being common between the two experiments. Further, keratin and immunoglobulins were also excluded from the analysis for quantitation. For these proteins, the ratio of the intensities of the reporter ions corresponding to CAD samples and the respective controls were compared. Proteins that had a ratio of either less than 0.8 or greater than 1.2 in at least three of the five groups (Group consists of CAD cases and the respective controls) were considered to be differentially expressed^24^. Using these criteria a total of 18 differentially expressed proteins were identified in the discovery phase (Table 2, Supplementary Table2). This excludes haemoglobin variants and the abundant proteins that were depleted from the plasma apart from keratin and immunoglobulins as mentioned earlier. Interestingly, proteins from the Apolipoprotein family (like Apo AI, Apo AIV, Apo B, Apo C1 and Apo CII) which are known to be involved in cholesterol transport were found to have lower expression in plasma of CAD patients than the respective controls (Table 2).

### Verification and Validation Phase

One of the important aspects of potential biomarker discovery from any cross sectional study is to verify and validate the findings of the discovery phase results in a larger sample size since the plasma proteomics workflow apart from the enormous high throughput power of exploring the proteome also has its own limitations. Therefore, further screening of the proteins that were found to be differentially expressed in the discovery phase was performed in another 40 samples (20 cases and 20 controls) in the verification (2^nd^) phase. Apart from these proteins, the four abundant proteins that were depleted in the first phase (apart from IgG and IgA) were also included in the 2^nd^ phase. All the 18 proteins that were differentially expressed in the discovery phase were screened in the verification phase. Thus, in this phase, the expressions of 18 different protein markers along with four proteins which were depleted prior to iTRAQ analysis were assayed. Of the 22 proteins included in the 2^nd^ phase 10 were significantly differentially expressed among controls and CAD patients (Table 3). Interestingly, albumin, the most abundant protein in the plasma, which was depleted and hence not considered in the discovery phase, was found to be significantly down regulated in CAD cases. The proteins that were found to be significantly differentially expressed in the 2^nd^ phase were then validated in the 3^rd^ phase in 506 samples (253 Cases and 253 Controls). Although Apo B was significantly down regulated in the 2^nd^ phase, it was not considered for the validation phase since it is known that statin decreases the levels of Apo B^25^,^26^ and most of the CAD cases in our study were consuming statins. In fact, the lower expression of ApoB that was found in both the 1^st^ and the 2^nd^ phase lends credence to our results. However, Apo CI was included in the validation phase since it plays an important role in cholesterol efflux from tissues akin to Apo AIV and Apo AI and also had a lower expression in the CAD patients, albeit not statistically significant. Thus, the levels of 8 protein markers (Apo AI, Albumin, Apo AIV, Adiponectin, Serum Amyloid P component, Glutathione peroxidase 3, Peroxiredoxin 2, and Apo C1) were measured in these samples using ELISA or Biochemical analyser. All the eight proteins were found to have significantly lower levels in the plasma of CAD patients (Table 4).

**Figure 2:**
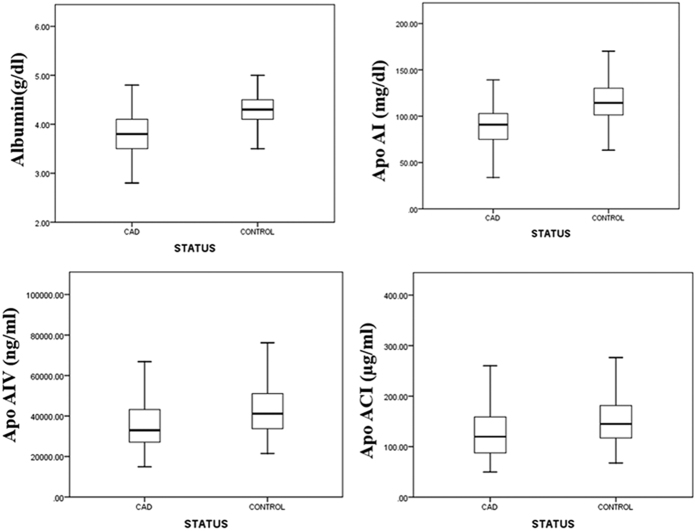
Significantly (p < 0.0001) differentially expressed protein levels after logistic regression analysis.

**Figure 3:**
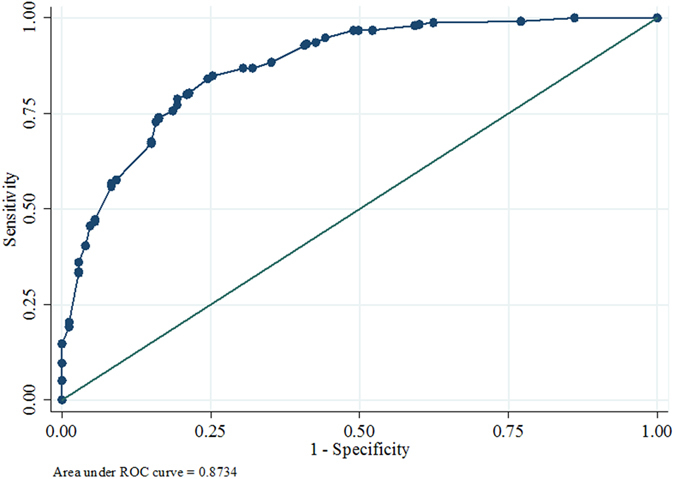
A ROC curve showing area under the curve 0.8734 for CAD cases.

Finally, a rigorous multivariate statistical analysis was performed to generate a model that could best discriminate CAD cases from controls. A multivariate logistic regression analysis was employed adjusting for age, sex, diet, diabetes, hypertension and smoking for all the samples. After the logistic regression analysis the levels of four proteins along with hypertension and diabetes remained significantly different in CAD cases (Table 5). The maximum odds ratio was found out to be 6.70 for albumin (p < 0.0001), followed by Apo AI (5.07, p < 0.0001), Apo CI (4.03, p = 0.001), and Apo AIV (2.63, p = 0.003) (Fig. 2). A ROC curve was generated and the area under the curve was found to be 0.8734 (Fig. 3) thus indicating that these protein markers have a high predictive value for CAD. Further a risk scale based score was also calculated upon this model and the score wise risk of the disease (probability %) is shown in Table 6. The probability of an individual with a risk score <1 to be a CAD case is 2.4% as compared to 98.2% probability of having CAD among those with a risk score of ≥7.0. These scores will be useful as a diagnostic aid for CAD. Since in general CAD is a late onset disease, to generate more insight we further segregated our subjects as young (<45 years old) and old (>45 years old) CAD cases and controls (Tables 7 and 8). All the four robust markers (Albumin, Apolipoprotein AI, Apolipoprotein AIV and Apolipoprotein C-I) from the previous analysis remain significant in both the groups compared to their respective controls. Interestingly, after multivariate logistic regression analysis we found two new markers peroxiredoxin-2 and dermicidin to be significantly associated with older CAD cases.

## Discussion

In this study we identified a panel of potential plasma protein markers for coronary artery disease using iTRAQ based proteomics workflow. Evolution of proteomic technologies has enabled researchers to explore newer disease markers in an unbiased manner^12^. This technique has been extensively used to identify markers for various diseases like cancer, sepsis, infectious diseases along with cardiovascular diseases^27^,^28^,^29^. Although high throughput plasma proteomic techniques hold a lot of promise in identifying potential markers, but, it is fraught with various challenges^30^. The pre-analytical variables like transportation, storage temperature and archival time have a large influence on the outcome of the results^31^. It is thus important that the case control samples should be collected and stored around the same time period. In this study we have taken care of all these issues. Depletion of high abundant proteins for the mining of low abundant proteins in plasma using proteomics is a standard practice in the field. Although we and others have shown that depletion results in loss of proteins that are bound to high abundant proteins like albumin, it acts almost as a necessary evil to enable the identification of low abundant proteins^30^. Thus, like all the studies that use depletion strategy, this study also will fail to identify the proteins that are bound to the abundant proteins and hence are virtually removed from the depleted plasma used in the proteomics experiments. However, we have included the four depleted proteins in the verification phase. Thus, in this study we have used the ability of iTRAQ technique to simultaneously analyse 8 different samples and followed it up with verification and validation phase to assess the robustness of the potential markers identified in the proteomics experiment.

Our results indicate that a panel of four proteins Apo AI, Apo AIV, Apo CI and albumin, are significantly associated with CAD even after multivariate logistic regression adjusting for various confounding factors. Since statins are known to have an effect on ApoB levels we did not evaluate the levels of ApoB in the validation phase since several of the CAD patients recruited in our study were on statins. However, to the best of our knowledge there are no reports suggesting that statins lower the levels of the other apolipoproteins found in our study. Despite this, we compared the levels of these 4 potential markers in CAD patients who were not on statins and controls. We found that even without statins, Albumin, apolipoprotein AI, apolipoprotein AIV, apolipoprotein CI were significantly downregulated in CAD patients (supplementary Table 3).

A recent study using Apo E knockout mice identified a panel of differentially expressed plasma proteins that are associated with the pathogenesis of CAD and that study also reported Apo AI to be downregulated both in 6 week and 12 week old knockout mouse compared to control. The INTERHEART study clearly showed that the levels of Apo AI are a better predictor than HDl-C for risk of acute myocardial infarction (AMI) in south Asians^32^. Interestingly, the median levels of Apo AI in South Asians (114.38 mg/dL) is far lower than the median levels in western population (130.5 mg/dL)^32^,^33^. Similarly, studies have shown that the levels of Apo AIV is lower in men with CAD^34^. Studies in mice have shown that overexpression of Apo AIV and Apo AI increases the cholesterol efflux, endogenous cholesterol esterification rates and HDL associated Platelet-activating factor acetylhydrolase activity in these animals. Further, the antioxidant property of apo AI and AIV probably helps in inhibition of LDL oxidation which is one of the key mechanisms for atherosclerosis progression^35^,^36^,^37^. The role of these two proteins along with Apo C1 is known largely for cholesterol efflux and inhibition of cholesteryl ester transfer proteins activity^38^,^39^. The intermittent lipid efflux has been shown to be related with the progression of atherosclerosis. The extent of cholesterol efflux from tissues by HDL plays a vital role in maintaining lipid homeostasis. Thus, the low expression of these Apo lipoproteins which are major component of HDL in the blood plasma of coronary artery disease patients’ indicates the altered state of lipid metabolism and impaired cholesterol efflux. In fact, newer therapeutic approaches have been developed with Apo AI mimetic peptides which have excellent potential to promote atherosclerosis regression^40^.

One of the important findings in our study was the status of albumin in CAD patients. Our observation that the albumin levels are significantly low is supported by a meta-analysis of eight prospective population based studies of albumin and coronary heart disease before 1998^41^. The meta-analysis confirmed that the levels of albumin were significantly down-regulated in coronary heart disease (38 g/L in CAD and 42 g/L in Controls). Microalbuminuria has also been associated with hypertension^42^. Since in our study both hypertension and low albumin levels were found to be associated with CAD, it would be tempting to assume that the low levels of albumin in CAD patients could be due to higher number of hypertensives. However, sub classification of our data on the basis of hypertensives and normotensives clearly indicate that albumin levels were not significantly associated with hypertension both in controls and in CAD cases (Supplementary Table 4). In fact there are studies clearly showing microalbuminuria could be an independent risk factor for cardiovascular disease even in individuals without hypertension^42^,^43^. Recently a study done in a Chinese population also reported that low serum albumin and globulin are independently associated with coronary heart disease^44^. Although the mechanism underlying the association of albumin with CAD is not clear there are several hypothesis proposed-the antioxidant capacity of albumin being one of them. A more plausible mechanism might be its role in cholesterol efflux. It has been clearly shown in a cell culture model that albumin can stimulate cholesterol efflux in a dose dependent manner^45^. It has also been shown that albumin promotes multidirectional transfer of cholesterol between cells and lipoproteins akin to HDL and since the concentration of albumin in plasma are extremely high it contributes to a significant proportion of cholesterol efflux^46^,^47^. Recently, Ishikawa *et al*. using isolated macrophages from CAD patients have demonstrated that cholesterol efflux capacity was the only significant predictor for CAD with an area under the curve of about 0.67. However, this study is limited by the fact that cholesterol efflux assay using macrophages isolated from large population are cumbersome and have practical limitations. On the contrary, our study demonstrated that multiple protein markers involved in RCT could be diagnosed easily in blood. The development of non-invasive biomarker panel from blood holds the key clinical implications for quick screening of populations such as Indians who are at high risk of CAD. Interestingly, we also found peroxiredoxin-2 and dermicidin to be significantly associated with older (>45 years) CAD cases. The levels of peroxiredoxin-2 and dermicidin was higher among young (<45 years ) CAD cases 23.75 ng/ml and 206.12 ng/ml respectively compared to 17.67 ng/ml and 193.19 ng/ml (p = 0.05, 0.3 respectively) in younger controls (<45 years). However, in the older CAD cases the levels of peroxiredoxin-2 and dermicidin remains significantly lower even after performing multivariate logistic regression adjusting for several confounding factors. It has been postulated that with ageing the response towards oxidative stress decreases which is in part reflected by the levels of peroxiredoxin-2 among younger and older CAD cases^48^,^49^. This finding also highlights the necessity of implementing age specific disease biomarkers for metabolic diseases such as cardiovascular and diabetes.

Thus, in this study, we have identified a panel of four proteins that along with hypertension and diabetes are significantly associated with CAD. Most of the proteins that we found to be low in CAD cases are involved in cholesterol efflux. The predictive statistical model was able to generate a Receiver Operating Characteristic (ROC) curve with an area under the curve of 0.8734 which is considered to be extremely significant. Further, we propose a risk score based on the panel of markers identified in this study that could be useful in predicting the likelihood of an individual to suffer from CAD. This scoring can be utilized in a clinical setup easily by determining the level of these markers. Our study also highlights the importance of considering the major plasma proteins at least in validation phase which are generally removed in a discovery phase proteomic experiment. However, the robustness of this panel of markers in terms of their predictive ability needs to be confirmed in a large prospective cohort for complex metabolic disorders such as CAD with a special emphasis on age. We would like to emphasize the importance of a multi marker approach compared to identification of single biomarker.

## Material and Methods

### Study design and patients groups

A total of 556 individuals (278 cases and 278 controls) of Indo-European origin mainly from Northern India were recruited for the whole study including discovery, verification and validation phase. All the stable CAD patients (confirmed after coronary angiography) were recruited from the Department of Cardiology, All India Institute of Medical Sciences (AIIMS), New Delhi, India. The controls of Indo-European origin were recruited from various parts of the National Capital Region. These individuals neither had a family history of cardiovascular disease nor had any sudden chest pain or discomfort. Further they were not having any difficulty in climbing up-to three floors. All the patients were diagnosed with multi-vessel stenosis. Patients with heart failure, MI, renal failure, atrial fibrillation or any other complications were excluded. Individuals below 18 years of age and pregnant women were excluded from the study. Written informed consent was obtained from the study participants before enrolment. The study was carried out in accordance with the Principles of the Helsinki Declaration and was approved by the ethics committee of both AIIMS and the CSIR-Institute of Genomics and Integrative Biology.

### Blood Plasma Isolation

Blood samples were collected in EDTA vacutainer from all the study participants. For CAD patients, the blood samples were taken after the coronary angiography was performed and the pathological status was confirmed at All India Institute of Medical Sciences, New Delhi (Dept. of Cardiology), New Delhi. The blood samples were stored upright at 4 °C until they were spun at 1300 × g at 4 °C for 15 minutes. Aliquots (500 μl) of the separated plasma were then stored at −80 °C for further analysis. To minimize the variations in the proteome profile this standard operating protocol was followed for all the cases and controls. It was ensured that the entire process-from collection of blood to storage at −80 °C was completed within 70–80 minutes for all the samples.

### Immunodepletion of High abundant protein and sample preparation

Immunodepletion of six most abundant proteins were done using multiple affinity removal (MARS- Hu-6) cartridge (Agilent, USA) following manufacturer’s protocol as described earlier^30^. This cartridge enables depletion of Albumin, IgG, Antitrypsin, IgA, Transferrin and Haptoglobin. Equal amount of protein from two samples were pooled to minimize intra-individual heterogeneity (Fig. 1). Depleted fractions were exchanged with 0.5 M TEAB (pH 8.5) buffer using 3 kd cut-off filter (Millipore, USA) and quantitated using Bradford assay.

### Protein reduction, alkylation, tryptic digestion, and iTRAQ labelling

Samples containing 50 μg of protein (at a concentration of 2.5 mg/ml) in 0.5 M TEAB (pH 8.5), were denatured with 1 μl of 2% (w/v) SDS (stock solution), reduced with freshly prepared DTT (Dithiotheritol; 25 mM) for 30 minutes (60 °C) and alkylated using IAA (Iodoacetamide; 55 mM) for 20 minutes in dark at room temperature^24^. It was then incubated with trypsin (Promega V511) at 37 °C at a ratio of 1:10 (trypsin to protein), for 16 hours. Digested samples were labelled with iTRAQ reagents following manufacturer’s protocol (Applied Biosystems, Foster City, CA). Two independent iTRAQ experiments were done, the first using a 8-plex reaction while the second using a 4-plex reaction. 50 μg of protein digest from each group was labelled with the respective iTRAQ reagents for 2 hours after which the reaction was quenched by adding 100 μl of milliQ water. The samples were then pooled and dried by centrifugal evaporation.

### Peptide fractionation with strong-cation-exchange (SCX) chromatography

iTRAQ labelled peptides were then fractionated by cation exchange chromatography using high performance liquid chromatography (Waters, Inc, Milford, MA) with a Zorbax 300 SCX column (5 μm; 2.1 mm × 150 mm, Agilent, USA). Lyophilized peptides were reconstituted in 2 ml (1 ml for 4-plex) loading buffer (buffer A consisting of 10 mM KH_2_PO_4_ in 75:25 water: acetonitrile, pH 2.9). The flow rate was kept at 0.4 ml/min for 17 minutes for loading after which it was increased to 0.8 ml/min. The following gradient program was employed: 0% Buffer B (10 mM KH_2_PO_4_, 1 M KCl in 75:25 water: acetonitrile (pH 2.7) for 17 min, 0% to 50% Buffer B in 39 min, 50% to 100% Buffer B in 5 min, 100% Buffer B for 10 min, 100% to 0% Buffer B in 8 min, and 0% Buffer B for 10 min (Supplementary Fig. 1). Eluting peptides were monitored at 214 nm and 20 fractions were collected using a fraction collector (FC 203B, Gilson). These fractions were then lyophilized by centrifugal evaporation (eppendorf).

### Reverse-phase liquid chromatography separation

Each of the fractions collected by cation exchange chromatography were further subjected to reverse phase chromatography using a nano- LC system (Tempo TM LC MALDI, USA), using a Chromolith Caprod RP-18e column (3 μm, 200 A°, 200 μm × 15 cm, MERCK) at a flow rate of 1 μl/min. A binary gradient with Solvent A (98% milliQ H_2_O, 2% ACN, and 0.1% formic acid) and Solvent B (2% milliQ H_2_O, 98% ACN, and 0.1% formic acid) was employed as the mobile phase. Prior to nano-LC separation, lyophilized SCX fractions were solubilized in 50 μl of Solvent A. 12 μl of sample were injected into a MicroTrap C18 cartridge (MICHROM Bioresources Inc, USA) for desalting using buffer A at a flow rate of 10μl/min for 45 minutes. After this, reverse phase separation was carried out over a period of 72 min, with the following gradient: Solvent B was ramped up from 3% to 7% in 5 min, then 7% to 25% over the next 30 minutes, 25% to 30% in another 5 minutes, 30% to 50% in next 5 minutes, 50% to 90% in next 3 minutes and continued with 90% for another 8 minutes to elute the highly retained species and then to 5% in 1 min (Supplementary Fig. 2). The LC-MALDI spotter was used to spot the peptides. The CHCA (α-Cyano-4-hydroxycinnamic acid) matrix (Bruker Daltonics), with a concentration of 5 mg/ml in 80% ACN, in milliQ water with 0.1% TFA, was continuously added to the column effluent at a flow rate of 1 μl/min via a syringe pump. Spotting was performed from 5–55 minutes during the LC separation at an interval of 7000 milli second per spot.

### Matrix assisted laser desorption ionization (MALDI) Mass spectrometry

The spotted plates from LC-MALDI were then subjected to MALDI MS/MS analysis using 5800 MALDI TOF/TOF (Applied Biosystems). MS spectra were acquired across the mass range of 800–4000 Da in reflector positive mode. The laser power was set at 3500 for MS with 1000 total shots per spectrum. Internal calibration was performed using standard calibration mix 5 (Applied Biosystems). The MS/MS spectra were acquired using 2 kV positive mode (CID- Collision induced dissociation; on) and the laser intensity was set at 4200. MS/MS spectra were acquired for the 25 most abundant precursor ions, with a total accumulation of 3000 shots. For MS/MS precursor selection, the minimum signal/noise ratio (S/N) filter was set at 25 with an exclusion list for CHCA matrix and keratin peaks^50^.

### Database searching

All MS and MS/MS spectra generated from LC-MALDI were submitted for database searching and quantitative analysis using Protein Pliot v 3.0 (Applied Biosystems). The searches were performed based on Paragon algorithm. The search parameters used for the plasma samples allowed for fixed modifications by IAA at cysteine residues and iTRAQ labeling, variable oxidation at methionine, and a single missed cleavage. A level of 1% FDR (False discovery rate) correction (≥2.0 unused score based on paragon algorithm) was applied to search the data in uniprot-human database.

### ELISA (Enzyme linked immunosorbent assay) and Biochemical analysis

Enzyme linked immune sorbent assays were performed according to the manufacturer’s protocol for the following proteins: Actin Cytoplasmic 2, Apo A IV, GPX3, Peroxiredoxin-2, Serum Amyloid P component, Fibrinogen gamma chain, Transferrin, Protein AMBP, Dermcidin, apolipoprotein CII, apolipoprotein L1, Pregnancy zone protein (Uscn Life Science Inc.); Apo CI, Adiponectin (AssayPro). The levels of Albumin, ApoB, Alpha 1-acid glycoprotein 2, alpha-1-antitrypsin, haptoglobin and Apo AI were determined using Biochemical Analyser (Roche, Cobas Integra). The assays for Thymosin beta 4 (Shanghai Bluegene Biotech Co, China) and vitronectin (Takara Bio Inc. Shiga, Japan) were also performed by ELISA based assays provided by the manufracturer.

### Statistical analysis

The plasma levels of each protein was compared between the cases and controls using ‘t’-test. Using ROC (Receiver operating characteristic) analysis best threshold value was determined for each protein. Taking that cut-off value as determined by ROC, odds ratios for CAD associated with each protein were calculated along with 95% CI. Multivariate logistic regression analysis was performed to identify the independent predictors of CAD. Based on the regression coefficients of the significant predictors identified in the stepwise multiple logistic regression analysis; a risk score was calculated. Predicted probability of CAD for various ranges of risk scores was calculated. A p value of less than 0.05 was considered significant. Analyses were performed using Stata 12.1 (StataCorp, College Station, Texas).

## Additional Information

**How to cite this article**: Basak, T. *et al*. Plasma proteomic analysis of stable coronary artery disease indicates impairment of reverse cholesterol pathway. *Sci. Rep.* **6**, 28042; doi: 10.1038/srep28042 (2016).

## Supplementary Material

Supplementary Information

## Figures and Tables

**Table 1:** Characteristics of samples used in this study.

**VARIABLES**	**DISCOVERY PHASE**		**VERIFICATION PHASE**		**VALIDATION PHASE**		
	**CAD (n** = **10)**	**CONTROL (n** = **10)**	**CAD (n** = **20)**	**CONTROL (n** = **20)**	**CAD (n** = **253)**	**CONTROL (n** = **253)**	p-VALUE*
HT-no.(%)	2(20)	2(20)	4(20)	1(5)	104(41.1)	38(15)	<0.0001
DM-no.(%)	2(20)	2(20)	6(30)	2(10)	78(30.8)	28(11)	<0.0001
SMOKING-no.(%)	Nil	Nil	6(30)	4(20)	114(45)	99(39.1)	0.177
DIET-veg.(%)	7(70)	9(90)	8(40)	9(45)	108(42.6)	121(47.8)	0.246
BMI (Kg/m^2^)	23.78 (22.0–24.85)	25.35 (21.76–27.11)	25.11 (23.3–28.28)	24.88 (22.43–28.8)	24.24 (22.57–26.52)	24.85 (22.58–27.43)	>0.05
STATIN-no.(%)	7(70)	0(0)	14(70)	0(0)	106(41.8)	0(0)	<0.0001
AGE-Mean(±SD)	56.25 ± 5.24	58.17 ± 4.21	50.05 ± 2.03	52.55 ± 5.10	51.74 ± 8.75	48.24 ± 10.36	<0.0001

n = number of individuals, Mean ± standard deviation reported for continuous variables while number (percentage) reported for discrete variables. HT- Hypertension, DM-Diabetes Mellitus, *p-value = calculated using chi-square or Mann-Whitney test.

**Table 2:** List of differentially expressed proteins from discovery phase analysis.

**Proteins**	**1st set (113:117)**	**2nd set (114:118)**	**3rd set (115:119)**	**4th set (114:116)**	**5th set (115:117)**
Actin, Cytoplasmic 2	1.6169	0.9357	1.4223	1.0331	2.4749
Adiponectin	0.683	1.2263	0.9485	0.7327	0.5968
Alpha-1-acid glycoprotein 2	0.7813	0.9165	0.5037	0.948	0.6942
AMBP protein	1.1621	1.3553	1.6125	1.532	0.7091
Apolipoprotein A-I	0.6278	1.0048	0.3461	0.457	0.9389
Apolipoprotein A-IV	0.7265	0.8253	0.6901	1.001	0.7471
Apolipoprotein B-100	0.7909	0.8079	0.7438	0.787	0.8872
Apolipoprotein C-I	0.7838	0.486	0.4974	0.2886	0.9913
Apolipoprotein C-II	1.5611	0.73	0.5236	0.3934	1.2617
Apolipoprotein L1	0.9343	0.7858	1.1346	0.6109	0.7831
Dermcidin	1.7836	1.4488	4.6536	1.1599	2.0635
Fibrinogen gamma chain	0.7686	1.1267	1.4741	1.4227	1.2637
Glutathione peroxidase 3	0.7084	1.2212	0.4706	0.7414	1.0727
Peroxiredoxin-2	1.3775	0.8013	2.1358	0.9135	1.2761
Pregnancy zone protein	1.2713	0.9843	1.2544	1.9943	1.5681
Serum amyloid P-component	0.6806	1.0889	0.3114	0.3759	0.7074
Thymosin beta-4	2.2765	1.5369	0.8007	2.4334	4.0036
Vitronectin	0.9495	1.4318	1.0016	1.2879	1.363

**Table 3:** Levels of selected candidates in Verification phase.

**Proteins**	**Cad (n = 20)**	**Control (n = 20)**	**Ratio**	**p value**
Alpha-1-acid glycoprotein 2 (g/L)	0.7 (0.5–0.89)	0.61 (0.57–0.8)	1.14	0.796
Apolipoprotein A-I (mg/dL)	97.39 (70.5–107.2)	115.49 (104.51–141.38)	0.8432	0.001
Apolipoprotein B (mg/dL)	51.56 (40.57–64.11)	87.25 (79.12–100.88)	0.59	<0.0001
Albumin (g/dL)	3.95 (3.62–4.27)	4.3 (4.12–4.5)	0.918	0.001
Alpha-1-antitrypsin (g/L)	1.21 (1.07–1.36)	1.2 (1.13–1.32)	1.008	0.547
Haptoglobin (g/L)	1.09 (0.79–1.55)	1.0 (0.6–1.35)	1.09	0.529
Apolipoprotein A-IV (ug/ml)	5.96 (3.69–13.17)	12.7 (9.49–18.59)	0.469	0.0045
Adiponectin (ng/ml)	6087.7 (4143.1–8469.46)	8690 (6887–11578)	0.7005	0.0025
Serum amyloid P-component (ng/ml)	37595 (21581–71477.8)	66672.7 (44503.3–100244.0)	0.563	0.028
Glutathione peroxidase 3 (μg/ml)	3.02 (1.71–3.93)	3.65 (2.87–4.86)	0.82	0.0338
Peroxiredoxin-2(ng/ml)	136.6 (125.2–159.5)	154.91(150.44–185.26)	0.881	0.0106
Apolipoprotein C-I (μg/ml)	112.95 (101.55–128.79)	124.71(113.2–138.62)	0.905	0.16
Actin cytoplasmic-2 (μg/ml)	3.59 (3.36–3.87)	3.91 (3.61–4.24)	0.918	0.056
Fibrinogen Gamma Chain (μg/ml)	5299.5 (968.6–9818.1)	5448.4 (291.3–9818.1)	0.972	0.88
Transferrin (mg/dL)	520.01 (119.6–1253.9)	515.25 (145.72–1310.2)	1.009	0.59
Vitronectin (μg/ml)	48.44 (14.40–99.06)	58.98 (8.84–103.02)	0.8212	0.88
Thymosin beta-4 (ng/ml)	4.48 (3.58–5.95)	4.44 (3.33–6.19)	1.009	0.685
Dermicidine(ng/ml)	28.27 (21–33.36)	23.67 (20.45–29.24)	1.19	0.137
ApoC2(μg/ml)	124.05 (91.71–229.99)	75.28 (49.28–106.48)	1.64	0.002
AMBP(mg/ml)	20.90 (17.30–25.23)	22.22 (16.02–27.57)	0.94	0.808
PZP (μg/ml)	34.43 (15.64–50.74)	55.96 (36.97–75.45)	0.615	0.010
Apol1 (μg/ml)	60.19 (47.47–90.41)	59.89 (22.85–73.04)	1.005	0.176

n = number of individuals, Median (Interquartile Range) reported, *p-value  = calculated using Mann-Whitney test.

**Table 4:** List of differentially expressed proteins in validation phase.

**Proteins**	**CAD (n** = **253)**	**Control (n** = **253)**	**RATIO**	**P -value**	**FUNCTION**
Adiponectin (ng/ml)	6771.27 (5038.24–10976.64)	8979.77 (6947.24–12666.71)	0.75	<0.0001	It is a chemokine which enhances glucose utilization and fatty acid combustion.
Apolipoprotein A-IV (μg/ml)	32947.32 (26952.50–43190.75)	41168.18 (33699.4–51181.45)	0.800	<0.0001	A major component of HDL and potential activator of LCAT
Apolipoprotein C-I (μg/ml)	119.75 (87.25–159.53)	144.99 (116.25–181.75)	0.825	<0.0001	It has important role in the interface of fatty acid uptake and major inhibitor of cholesteryl ester transfer protein (CETP)
Glutathione peroxidase 3 (ng/ml)	13599.26 (7474.89–18502.31)	16288.49 (10259.01–23961.43)	0.834	<0.0001	Plays protective role in oxidative damage
Peroxiredoxine-2 (ng/ml)	19.54 (11.30–29.62)	21.03 (13.68–84791.41)	0.929	0.002	It is involved in redox balance regulation; reduces peroxides via thioredoxin pathway
Serum amyloid P-component (ng/ml)	40855.06 (23281.44–69654.73)	47680.4 (27776.45–84791.41)	0.856	0.048	It is a acute phase protein and may functions as a calcium-dependent lectin
Albumin (g/dL)	3.8 (3.50–4.1)	4.3 (4.10–4.50)	0.88	<0.0001	It is the major transporter protein in plasma and has role in reverse cholesterol transport
Apolipoprotein A-I (mg/dL)	90.89 (74.98–102.87)	114.38 (101.14–130.31)	0.794	<0.0001	Major player in the process of reverse cholesterol transport and promotes cholesterol efflux
Apolipoprotein C-II (μg/ml)	147.12 (63.78–253.50)	129.18 (66.28–220.21)	1.138	0.138	Activator of lipoprotein lipase and a central player in lipoprotein metabolism
PZP(ng/ml)	46638.54 (22413.76–99311.91)	48318.57 (25560.9–96970.9)	0.965	0.384	It is a proteinase inhibitor and also has endopeptidase inhibitor activity

n = number of individuals, Median (Interquartile Range) reported, *p-value  = calculated using Mann-Whitney test.

**Table 5:** Significant parameters after multivariate logistic regression.

**Variables**	**Odds Ratio (95% CI)**	**p value**
Apolipoprotein AIV	2.63 (1.62–4.28)	<0.0001
Apolipoprotein C-I	4.03 (2.44–6.65)	<0.0001
Albumin	6.70 (3.96–11.32)	<0.0001
Apolipoprotein AI	5.07 (3.17–8.12)	<0.0001
Hypertension	2.76 (1.60–4.77)	<0.0001
Diabetes Mellitus	2.31 (1.27–4.22)	0.006

**Table 6:** Risks score corresponding to Probability (%) of CAD.

**Risk Score**	**Probability of CAD (%)**
<1.0	2.4
1–1.5	7.9
1.5–2	13.2
2–2.5	19.6
2.5–3	29.2
3–3.5	37.9
3.5–4	48.6
4–4.5	61.9
4.5–5	72.6
5–5.5	82.4
5.5–6	88.9
6–6.5	92.9
6.5–7	95.3
≥7	98.2

**Table 7:** Significant parameters after multivariate logistic regression for young (<45 years) CAD patients.

**Variables**	**p value**
Apolipoprotein AIV	<0.0001
Apolipoprotein C-I	0.006
Albumin	0.003
Apolipoprotein AI	<0.0001
Hypertension	0.002

**Table 8:** Significant parameters after multivariate logistic regression for old (>45 years) CAD patients.

**Variables**	**p value**
Apolipoprotein AIV	0.001
Apolipoprotein C-I	0.002
Albumin	<0.001
Apolipoprotein AI	<0.0001
Peroxiredoxin	0.001
Dermicidin	0.025
