# Supplementary material for: Plasma proteomic analysis of stable coronary artery disease indicates impairment of reverse cholesterol pathway
Source: Sci Rep. 2016 Jun 28;6:28042. doi: 10.1038/srep28042 (PMC4923873; doi:10.1038/srep28042)
Supplement: Supplementary Information [file srep28042-s1.pdf]

Supplementary information for Plasma proteomic analysis of stable coronary artery disease indicates impairment of reverse cholesterol pathway

Trayambak Basak [1,2], Vinay Singh Tanwar [1], Gourav Bharadwaj [1], Nitin Bhardwaj [1], Shadab Ahmad [1], Gaurav Garg [1], Sreenivas V [3], Ganesan Karthikeyan [4], Sandeep Seth [4], Shantanu Sengupta [1,2]

[1]Genomics and Molecular Medicine Unit, CSIR-Institute of Genomics and Integrative Biology, New Delhi

[2]Academy of Scientific & Innovative Research, New Delhi

[3]Department of Biostatistics, All India Institute of Medical Sciences, New Delhi

[4]Department of Cardiology, All India Institute of Medical Sciences, New Delhi

Supplementary Information:

Supplementary Figure 1: Representative SCX chromatography of 4-plex and 8 plex iTRAQ experiment

Supplementary Figure 2: Representative reverse phase chromatography of 4-plex and 8 plex iTRAQ experiment

Supplementary Table 1: List of proteins identified at 1% global protein FDR in 4-plex and 8-plex iTRAQ experiment

Supplementary Table 2: Discovery phase differentially expressed proteins with p value

Supplementary Table 3: Comparison of four markers (albumin, apolipoprotein aI, apolipoprotein aIV, apolipoprotein CI) among controls and CAD cases without statin therapy

Supplementary Table 4: Level of albumin among hypertensive and non-hypertensive controls and CAD cases

Supplementary figure 1

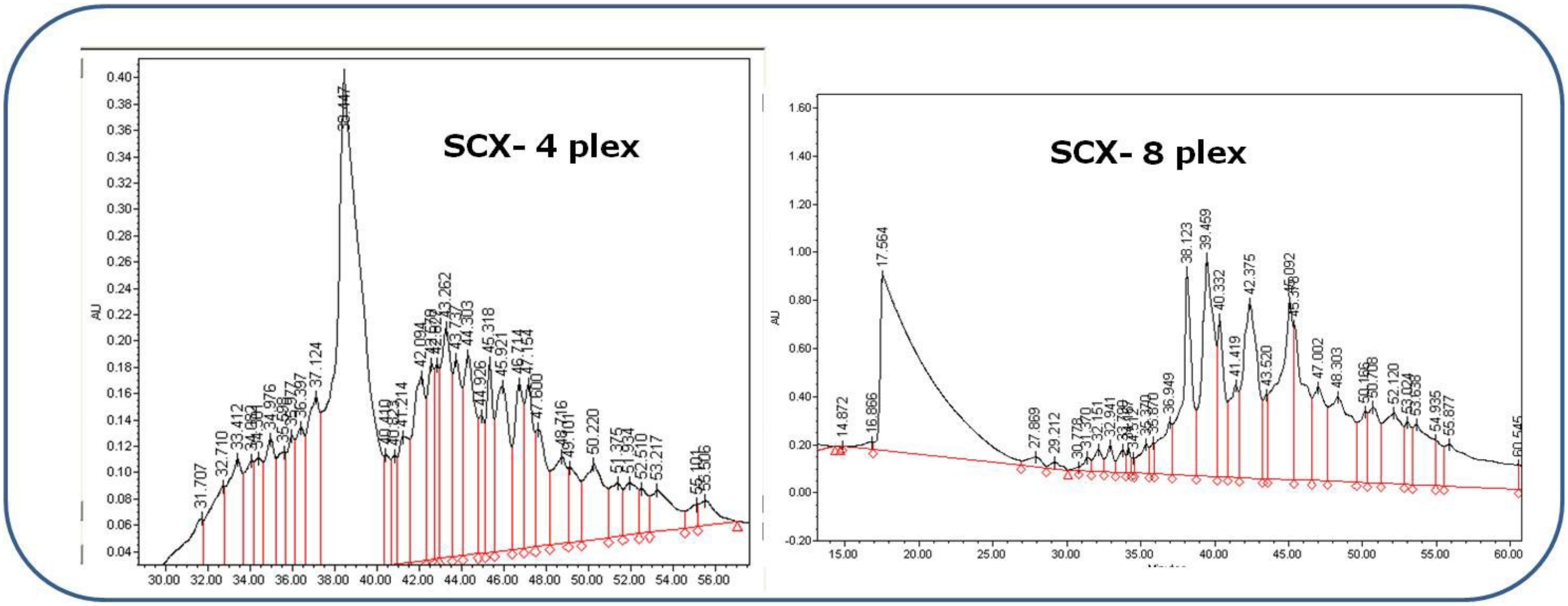

Supplementary figure 2

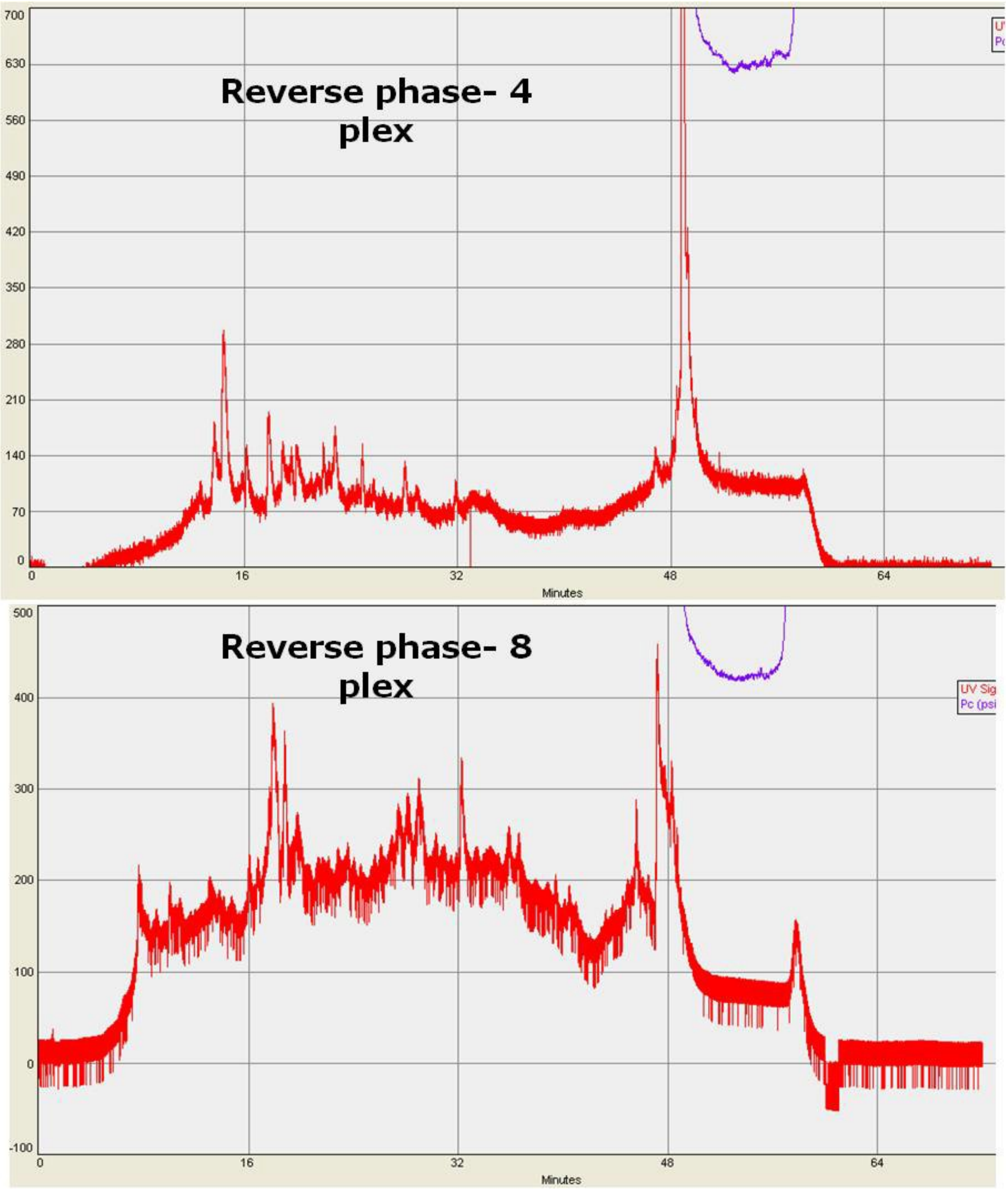

Supplementary table 1 (4plex experiment)

N	Unused	Total	% Cov	Accession #	Name	Species	Peptides(95%)
1	385.87	385.87	51.3	sp|P04114|APOB_HUMAN	Apolipoprotein B-100 OS=Homo sapiens GN=APOB PE=1 SV=1	HUMAN	255
2	296.8	296.8	72.6	sp|P01024|CO3_HUMAN	Complement C3 OS=Homo sapiens GN=C3 PE=1 SV=2	HUMAN	349
3	208.28	208.51	65.5	sp|P01023|A2MG_HUMAN	Alpha-2-macroglobulin OS=Homo sapiens GN=A2M PE=1 SV=1	HUMAN	274
4	161.97	164.39	52.1	sp|P0C0L5|CO4B_HUMAN	Complement C4-B OS=Homo sapiens GN=C4B PE=1 SV=1	HUMAN	129
5	141.51	141.51	46	sp|P02751|FINC_HUMAN	Fibronectin OS=Homo sapiens GN=FN1 PE=1 SV=3	HUMAN	111
6	139.71	139.72	81.1	sp|P02675|FIBB_HUMAN	Fibrinogen beta chain OS=Homo sapiens GN=FGB PE=1 SV=2	HUMAN	193
7	138.44	138.44	62.6	sp|P08603|CFAH_HUMAN	Complement factor H OS=Homo sapiens GN=CFH PE=1 SV=4	HUMAN	115
8	137.35	137.35	55.4	sp|P02671|FIBA_HUMAN	Fibrinogen alpha chain OS=Homo sapiens GN=FGA PE=1 SV=2	HUMAN	217
9	102.49	103.1	78.6	sp|P02679|FIBG_HUMAN	Fibrinogen gamma chain OS=Homo sapiens GN=FGG PE=1 SV=3	HUMAN	202
10	88.41	89.46	45.2	sp|P00450|CERU_HUMAN	Ceruloplasmin OS=Homo sapiens GN=CP PE=1 SV=1	HUMAN	90
11	86.91	87.57	34.1	sp|P01031|CO5_HUMAN	Complement C5 OS=Homo sapiens GN=C5 PE=1 SV=4	HUMAN	43
12	85.88	85.89	91.8	sp|P02647|APOA1_HUMAN	Apolipoprotein A-I OS=Homo sapiens GN=APOA1 PE=1 SV=1	HUMAN	182
13	83.61	83.61	65.7	sp|P00747|PLMN_HUMAN	Plasminogen OS=Homo sapiens GN=PLG PE=1 SV=2	HUMAN	54
14	80.81	80.81	60.2	sp|P02790|HEMO_HUMAN	Hemopexin OS=Homo sapiens GN=HPX PE=1 SV=2	HUMAN	99
15	79.67	79.67	41.4	sp|P00751|CFAB_HUMAN	Complement factor B OS=Homo sapiens GN=CFB PE=1 SV=2	HUMAN	62
16	71.73	71.73	68.9	sp|P06727|APOA4_HUMAN	Apolipoprotein A-IV OS=Homo sapiens GN=APOA4 PE=1 SV=3	HUMAN	58
17	69.67	69.67	57.8	sp|P02768|ALBU_HUMAN	Serum albumin OS=Homo sapiens GN=ALB PE=1 SV=2	HUMAN	48
18	69.11	69.17	61.4	sp|P02774|VTDB_HUMAN	Vitamin D-binding protein OS=Homo sapiens GN=GC PE=1 SV=1	HUMAN	63
19	66.18	66.42	60.6	sp|P00734|THRB_HUMAN	Prothrombin OS=Homo sapiens GN=F2 PE=1 SV=2	HUMAN	66
20	60.53	61.42	51.3	sp|P01008|ANT3_HUMAN	Antithrombin-III OS=Homo sapiens GN=SERPINC1 PE=1 SV=1	HUMAN	57
21	56.04	56.19	47.7	sp|P01042|KNG1_HUMAN	Kininogen-1 OS=Homo sapiens GN=KNG1 PE=1 SV=2	HUMAN	40
22	52.84	52.85	36.6	sp|Q14624|ITIH4_HUMAN	Inter-alpha-trypsin inhibitor heavy chain H4 OS=Homo sapiens GN=ITIH4 PE=1 SV=3	HUMAN	39
23	52.07	52.07	38.7	sp|P02765|FETUA_HUMAN	Alpha-2-HS-glycoprotein OS=Homo sapiens GN=AHSG PE=1 SV=1	HUMAN	60
24	51.35	51.4	31.6	sp|P19823|ITIH2_HUMAN	Inter-alpha-trypsin inhibitor heavy chain H2 OS=Homo sapiens GN=ITIH2 PE=1 SV=1	HUMAN	52
25	50.64	51.64	38.9	sp|P43652|AFAM_HUMAN	Afamin OS=Homo sapiens GN=AFM PE=1 SV=1	HUMAN	40
26	48.68	49.25	40.5	sp|P06396|GELS_HUMAN	Gelsolin OS=Homo sapiens GN=GSN PE=1 SV=1	HUMAN	32
27	45.42	45.47	52	sp|P01011|AACT_HUMAN	Alpha-1-antichymotrypsin OS=Homo sapiens GN=SERPINA3 PE=1 SV=2	HUMAN	50
28	43.12	43.12	33.8	sp|P13671|CO6_HUMAN	Complement component C6 OS=Homo sapiens GN=C6 PE=1 SV=3	HUMAN	25
29	42.29	42.3	46.3	sp|P02787|TRFE_HUMAN	Serotransferrin OS=Homo sapiens GN=TF PE=1 SV=2	HUMAN	23
30	42.01	42.65	34.4	sp|P19827|ITIH1_HUMAN	Inter-alpha-trypsin inhibitor heavy chain H1 OS=Homo sapiens GN=ITIH1 PE=1 SV=3	HUMAN	35
31	41.74	41.76	56.9	sp|P01009|A1AT_HUMAN	Alpha-1-antitrypsin OS=Homo sapiens GN=SERPINA1 PE=1 SV=3	HUMAN	29
32	38.98	38.98	37.3	sp|P10643|CO7_HUMAN	Complement component C7 OS=Homo sapiens GN=C7 PE=1 SV=2	HUMAN	22
33	37.4	37.4	45.3	sp|P02763|A1AG1_HUMAN	Alpha-1-acid glycoprotein 1 OS=Homo sapiens GN=ORM1 PE=1 SV=1	HUMAN	59
34	36.7	36.7	38.7	sp|P00736|C1R_HUMAN	Complement C1r subcomponent OS=Homo sapiens GN=C1R PE=1 SV=2	HUMAN	21
35	36.14	36.14	41.5	sp|P04196|HRG_HUMAN	Histidine-rich glycoprotein OS=Homo sapiens GN=HRG PE=1 SV=1	HUMAN	26
36	34.63	34.66	34.3	sp|P03952|KLKB1_HUMAN	Plasma kallikrein OS=Homo sapiens GN=KLKB1 PE=1 SV=1	HUMAN	20
37	34.53	34.53	35.2	sp|P02748|CO9_HUMAN	Complement component C9 OS=Homo sapiens GN=C9 PE=1 SV=2	HUMAN	23
38	33.96	33.96	46.1	sp|P25311|ZA2G_HUMAN	Zinc-alpha-2-glycoprotein OS=Homo sapiens GN=AZGP1 PE=1 SV=1	HUMAN	28
39	33.75	33.75	63.1	sp|P00738|HPT_HUMAN	Haptoglobin OS=Homo sapiens GN=HP PE=1 SV=1	HUMAN	18
40	33.62	33.62	50.1	sp|P10909|CLUS_HUMAN	Clusterin OS=Homo sapiens GN=CLU PE=1 SV=1	HUMAN	23
41	32.74	32.74	56.2	sp|P02749|APOH_HUMAN	Beta-2-glycoprotein 1 OS=Homo sapiens GN=APOH PE=1 SV=3	HUMAN	28
42	32.67	32.67	35.4	sp|P05155|IC1_HUMAN	Plasma protease C1 inhibitor OS=Homo sapiens GN=SERPING1 PE=1 SV=2	HUMAN	30
43	30.97	31.7	39.3	sp|P08697|A2AP_HUMAN	Alpha-2-antiplasmin OS=Homo sapiens GN=SERPINF2 PE=1 SV=3	HUMAN	19
44	29.81	29.82	33.7	sp|P07358|CO8B_HUMAN	Complement component C8 beta chain OS=Homo sapiens GN=C8B PE=1 SV=3	HUMAN	19
45	28.59	28.59	58.7	sp|P02649|APOE_HUMAN	Apolipoprotein E OS=Homo sapiens GN=APOE PE=1 SV=1	HUMAN	17
46	28.52	28.52	36.2	sp|P04004|VTNC_HUMAN	Vitronectin OS=Homo sapiens GN=VTN PE=1 SV=1	HUMAN	21
47	28.13	28.14	32.8	sp|P05156|CFAI_HUMAN	Complement factor I OS=Homo sapiens GN=CFI PE=1 SV=1	HUMAN	16
48	25.98	26.08	32.1	sp|P05546|HEP2_HUMAN	Heparin cofactor 2 OS=Homo sapiens GN=SERPIND1 PE=1 SV=3	HUMAN	21
49	25.26	25.26	42.3	sp|P36955|PEDF_HUMAN	Pigment epithelium-derived factor OS=Homo sapiens GN=SERPINF1 PE=1 SV=3	HUMAN	13
50	25.08	25.08	30.7	sp|P35858|ALS_HUMAN	Insulin-like growth factor-binding protein complex acid labile chain OS=Homo sapiens GN=IGFA	HUMAN	13
51	24.77	24.77	28.3	sp|P04217|A1BG_HUMAN	Alpha-1B-glycoprotein OS=Homo sapiens GN=A1BG PE=1 SV=3	HUMAN	22
52	23.48	23.48	63	sp|P02652|APOA2_HUMAN	Apolipoprotein A-II OS=Homo sapiens GN=APOA2 PE=1 SV=1	HUMAN	26
53	22.95	22.95	26.8	sp|P01019|ANGT_HUMAN	Angiotensinogen OS=Homo sapiens GN=AGT PE=1 SV=1	HUMAN	13
54	22.24	22.25	40.1	sp|P29622|KAIN_HUMAN	Kallistatin OS=Homo sapiens GN=SERPINA4 PE=1 SV=3	HUMAN	10
55	22.22	22.65	31.5	sp|P05160|F13B_HUMAN	Coagulation factor XIII B chain OS=Homo sapiens GN=F13B PE=1 SV=3	HUMAN	13
56	22.03	22.03	39.7	sp|P63261|ACTG_HUMAN	Actin, cytoplasmic 2 OS=Homo sapiens GN=ACTG1 PE=1 SV=1	HUMAN	11
57	21.52	21.52	43.3	cont|000141	spt|P00761| Trypsin precursor (EC 3.4.21.4) [Sus scrofa (contaminant)]	Sus scrofa	29
58	20.94	20.96	25.9	sp|P07225|PROS_HUMAN	Vitamin K-dependent protein S OS=Homo sapiens GN=PROS1 PE=1 SV=1	HUMAN	11
59	20.82	22.83	17.3	sp|P06681|CO2_HUMAN	Complement C2 OS=Homo sapiens GN=C2 PE=1 SV=2	HUMAN	13
60	20.6	20.6	33.1	cont|000135	cra|hCP1609934.2| keratin 1 (epidermolytic hyperkeratosis) [Homo sapiens (contaminant)]	Homo sapi	10
61	20.27	20.27	20.1	sp|P80108|PHLD_HUMAN	Phosphatidylinositol-glycan-specific phospholipase D OS=Homo sapiens GN=GPLD1 PE=1 SV=3	HUMAN	10
62	20.01	20.01	34.7	sp|P27169|PON1_HUMAN	Serum paraoxonase/arylesterase 1 OS=Homo sapiens GN=PON1 PE=1 SV=2	HUMAN	12
63	19.79	19.79	23.3	sp|P09871|C1S_HUMAN	Complement C1s subcomponent OS=Homo sapiens GN=C1S PE=1 SV=1	HUMAN	10
64	19.59	19.59	28.6	sp|P07357|CO8A_HUMAN	Complement component C8 alpha chain OS=Homo sapiens GN=C8A PE=1 SV=2	HUMAN	12
65	18.64	18.64	46.6	sp|P05090|APOD_HUMAN	Apolipoprotein D OS=Homo sapiens GN=APOD PE=1 SV=1	HUMAN	11
66	18.02	27.72	42.3	sp|P19652|A1AG2_HUMAN	Alpha-1-acid glycoprotein 2 OS=Homo sapiens GN=ORM2 PE=1 SV=2	HUMAN	39
67	17.86	17.98	10.8	sp|P04275|VWF_HUMAN	von Willebrand factor OS=Homo sapiens GN=VWF PE=1 SV=2	HUMAN	8
68	17.47	17.52	19.7	sp|Q06033|ITIH3_HUMAN	Inter-alpha-trypsin inhibitor heavy chain H3 OS=Homo sapiens GN=ITIH3 PE=1 SV=2	HUMAN	11
69	17.45	17.45	37.7	sp|P02743|SAMP_HUMAN	Serum amyloid P-component OS=Homo sapiens GN=APCS PE=1 SV=2	HUMAN	14
70	17.42	17.42	29.4	sp|P02750|A2GL_HUMAN	Leucine-rich alpha-2-glycoprotein OS=Homo sapiens GN=LRG1 PE=1 SV=2	HUMAN	10
71	17.41	17.41	28.4	sp|Q96IY4|CBPB2_HUMAN	Carboxypeptidase B2 OS=Homo sapiens GN=CPB2 PE=1 SV=1	HUMAN	11
72	17.17	17.17	25.3	sp|P04003|C4BPA_HUMAN	C4b-binding protein alpha chain OS=Homo sapiens GN=C4BPA PE=1 SV=2	HUMAN	8
73	17	17	38.4	sp|P02760|AMBP_HUMAN	Protein AMBP OS=Homo sapiens GN=AMBP PE=1 SV=1	HUMAN	12
74	16.09	16.09	56.7	sp|P02753|RET4_HUMAN	Retinol-binding protein 4 OS=Homo sapiens GN=RBP4 PE=1 SV=3	HUMAN	19
75	16.03	16.03	34.9	sp|Q96PD5|PGRP2_HUMAN	N-acetylmuramoyl-L-alanine amidase OS=Homo sapiens GN=PGLYRP2 PE=1 SV=1	HUMAN	9
76	15.8	15.8	25.6	sp|Q16610|ECM1_HUMAN	Extracellular matrix protein 1 OS=Homo sapiens GN=ECM1 PE=1 SV=2	HUMAN	8
77	15.8	15.8	60.9	sp|P07360|CO8G_HUMAN	Complement component C8 gamma chain OS=Homo sapiens GN=C8G PE=1 SV=3	HUMAN	10
78	15.23	15.23	28.1	sp|P01871|IGHM_HUMAN	Ig mu chain C region OS=Homo sapiens GN=IGHM PE=1 SV=3	HUMAN	8
79	14	14.14	55.4	sp|P02654|APOC1_HUMAN	Apolipoprotein C-I OS=Homo sapiens GN=APOC1 PE=1 SV=1	HUMAN	8
80	13.98	14.11	24.9	sp|P51884|LUM_HUMAN	Lumican OS=Homo sapiens GN=LUM PE=1 SV=2	HUMAN	10
81	13.32	13.34	53.1	sp|P35542|SAA4_HUMAN	Serum amyloid A-4 protein OS=Homo sapiens GN=SAA4 PE=1 SV=1	HUMAN	12
82	13.23	13.64	21.7	sp|Q04756|HGFA_HUMAN	Hepatocyte growth factor activator OS=Homo sapiens GN=HGFAC PE=1 SV=1	HUMAN	6
83	12.67	12.67	26.1	sp|P17936|IBP3_HUMAN	Insulin-like growth factor-binding protein 3 OS=Homo sapiens GN=IGFBP3 PE=1 SV=2	HUMAN	6
84	12.5	12.52	68	sp|P68871|HBB_HUMAN	Hemoglobin subunit beta OS=Homo sapiens GN=HBB PE=1 SV=2	HUMAN	6
85	12.46	12.46	23.9	sp|P00740|FA9_HUMAN	Coagulation factor IX OS=Homo sapiens GN=F9 PE=1 SV=2	HUMAN	6
86	12.38	12.38	28.4	sp|P01860|IGHG3_HUMAN	Ig gamma-3 chain C region OS=Homo sapiens GN=IGHG3 PE=1 SV=2	HUMAN	6

87	12.35	14.35	23.1	sp|P05543|THBG_HUMAN	Thyroxine-binding globulin OS=Homo sapiens GN=SERPINA7 PE=1 SV=2	HUMAN	8
88		12.22	21.1	sp|P00748|FA12_HUMAN	Coagulation factor XII OS=Homo sapiens GN=F12 PE=1 SV=2	HUMAN	6
89	11.9	11.9	17.1	sp|P22792|CPN2_HUMAN	Carboxypeptidase N subunit 2 OS=Homo sapiens GN=CPN2 PE=1 SV=2	HUMAN	5
90	11.85	11.85	32.5	sp|O95445|APOM_HUMAN	Apolipoprotein M OS=Homo sapiens GN=APOM PE=1 SV=2	HUMAN	6
91	11.79	11.79	27.5	sp|P02746|C1QB_HUMAN	Complement C1q subcomponent subunit B OS=Homo sapiens GN=C1QB PE=1 SV=2	HUMAN	15
92	11.52	11.92	24.4	sp|O14791|APOL1_HUMAN	Apolipoprotein L1 OS=Homo sapiens GN=APOL1 PE=1 SV=5	HUMAN	6
93	10.88	10.88	15.5	sp|P06276|CHLE_HUMAN	Cholinesterase OS=Homo sapiens GN=BCHE PE=1 SV=1	HUMAN	5
94	10.57	10.57	38.5	sp|O75636|FCN3_HUMAN	Ficolin-3 OS=Homo sapiens GN=FCN3 PE=1 SV=2	HUMAN	6
95	10.56	10.56	55.5	sp|P05452|TETN_HUMAN	Tetranectin OS=Homo sapiens GN=CLEC3B PE=1 SV=2	HUMAN	5
96	10.27	33.74	23.2	sp|P20742|PZP_HUMAN	Pregnancy zone protein OS=Homo sapiens GN=PZP PE=1 SV=3	HUMAN	33
97	10.11	10.11	59.4	sp|P02655|APOC2_HUMAN	Apolipoprotein C-II OS=Homo sapiens GN=APOC2 PE=1 SV=1	HUMAN	7
98	9.91	9.91	25.4	sp|P27918|PROP_HUMAN	Properdin OS=Homo sapiens GN=CFP PE=1 SV=2	HUMAN	5
99	9.7	9.7	34.7	sp|P31151|S10A7_HUMAN	Protein S100-A7 OS=Homo sapiens GN=S100A7 PE=1 SV=4	HUMAN	6
100	9.65	12.03	14.3	sp|P12259|FA5_HUMAN	Coagulation factor V OS=Homo sapiens GN=F5 PE=1 SV=3	HUMAN	5
101	9.1	9.1	38	sp|P69905|HBA_HUMAN	Hemoglobin subunit alpha OS=Homo sapiens GN=HBA1 PE=1 SV=2	HUMAN	5
102	8.49	8.49	33.1	sp|P02747|C1QC_HUMAN	Complement C1q subcomponent subunit C OS=Homo sapiens GN=C1QC PE=1 SV=3	HUMAN	9
103	8.48	10.49	10.9	sp|P00742|FA10_HUMAN	Coagulation factor X OS=Homo sapiens GN=F10 PE=1 SV=2	HUMAN	5
104	8.29	8.29	51.8	sp|P06702|S10A9_HUMAN	Protein S100-A9 OS=Homo sapiens GN=S100A9 PE=1 SV=1	HUMAN	4
105	8.16	8.24	31.3	sp|P05154|IPSP_HUMAN	Plasma serine protease inhibitor OS=Homo sapiens GN=SERPINA5 PE=1 SV=2	HUMAN	5
106	8.14	8.22	28.7	sp|P00915|CAH1_HUMAN	Carbonic anhydrase 1 OS=Homo sapiens GN=CA1 PE=1 SV=2	HUMAN	4
107	8.07	8.77	13.8	sp|P35527|K1C9_HUMAN	Keratin, type I cytoskeletal 9 OS=Homo sapiens GN=KRT9 PE=1 SV=2	HUMAN	4
108	8	10	17.5	sp|Q14520|HABP2_HUMAN	Hyaluronan-binding protein 2 OS=Homo sapiens GN=HABP2 PE=1 SV=1	HUMAN	5
109	7.46	11.78	26	sp|P08519|APOA_HUMAN	Apolipoprotein(a) OS=Homo sapiens GN=LPA PE=1 SV=1	HUMAN	7
110	7.44	11.16	14.1	sp|Q92954|PRG4_HUMAN	Proteoglycan 4 OS=Homo sapiens GN=PRG4 PE=1 SV=2	HUMAN	5
111	7.4	9.4	15	sp|P03951|FA11_HUMAN	Coagulation factor XI OS=Homo sapiens GN=F11 PE=1 SV=1	HUMAN	5
112	7.31	7.31	9.8	sp|P43251|BTD_HUMAN	Biotinidase OS=Homo sapiens GN=BTD PE=1 SV=1	HUMAN	4
113	6.85	6.85	53.1	sp|P02766|TTHY_HUMAN	Transthyretin OS=Homo sapiens GN=TTR PE=1 SV=1	HUMAN	4
114	6.71	6.71	24.3	sp|P22352|GPX3_HUMAN	Glutathione peroxidase 3 OS=Homo sapiens GN=GPX3 PE=1 SV=2	HUMAN	3
115	6.6	6.6	27.7	sp|P00746|CFAD_HUMAN	Complement factor D OS=Homo sapiens GN=CFD PE=1 SV=5	HUMAN	3
116	6.43	7.15	15.4	sp|P13645|K1C10_HUMAN	Keratin, type I cytoskeletal 10 OS=Homo sapiens GN=KRT10 PE=1 SV=4	HUMAN	3
117	6.32	6.33	15	sp|P00488|F13A_HUMAN	Coagulation factor XIII A chain OS=Homo sapiens GN=F13A1 PE=1 SV=4	HUMAN	3
118	6.24	6.24	23	sp|Q15848|ADIPO_HUMAN	Adiponectin OS=Homo sapiens GN=ADIPOQ PE=1 SV=1	HUMAN	3
119	6.2	6.2	48.7	sp|P62988|UBIQ_HUMAN	Ubiquitin OS=Homo sapiens GN=RPS27A PE=1 SV=1	HUMAN	3
120	6.11	14.79	37	sp|Q03591|FHR1_HUMAN	Complement factor H-related protein 1 OS=Homo sapiens GN=CFHR1 PE=1 SV=2	HUMAN	12
121	6.11	6.21	12.8	sp|P23142|FBLN1_HUMAN	Fibulin-1 OS=Homo sapiens GN=FBLN1 PE=1 SV=4	HUMAN	4
122	6.04	6.33	15.6	sp|P04070|PROC_HUMAN	Vitamin K-dependent protein C OS=Homo sapiens GN=PROC PE=1 SV=1	HUMAN	3
123	6.02	6.02	48.2	sp|P81605|DCD_HUMAN	Dermcidin OS=Homo sapiens GN=DCD PE=1 SV=2	HUMAN	3
124	6	6	25.2	sp|P55056|APOC4_HUMAN	Apolipoprotein C-IV OS=Homo sapiens GN=APOC4 PE=1 SV=1	HUMAN	3
125	6	6	20.8	sp|P02745|C1QA_HUMAN	Complement C1q subcomponent subunit A OS=Homo sapiens GN=C1QA PE=1 SV=2	HUMAN	5
126	6	6	51.5	sp|P02656|APOC3_HUMAN	Apolipoprotein C-III OS=Homo sapiens GN=APOC3 PE=1 SV=1	HUMAN	9
127	5.7	10.07	12.7	sp|Q9NZP8|C1RL_HUMAN	Complement C1r subcomponent-like protein OS=Homo sapiens GN=C1RL PE=1 SV=2	HUMAN	6
128	5.53	5.53	19.5	sp|P08571|CD14_HUMAN	Monocyte differentiation antigen CD14 OS=Homo sapiens GN=CD14 PE=1 SV=2	HUMAN	3
129	5.5	5.54	24.2	sp|P49908|SEPP1_HUMAN	Selenoprotein P OS=Homo sapiens GN=SEPP1 PE=1 SV=3	HUMAN	3
130	5.5	5.52	15.2	sp|P18428|LBP_HUMAN	Lipopolysaccharide-binding protein OS=Homo sapiens GN=LBP PE=1 SV=3	HUMAN	3
131	5.48	5.49	16.8	sp|P15169|CBPN_HUMAN	Carboxypeptidase N catalytic chain OS=Homo sapiens GN=CPN1 PE=1 SV=1	HUMAN	2
132	5.23	5.23	20.2	sp|P32119|PRDX2_HUMAN	Peroxiredoxin-2 OS=Homo sapiens GN=PRDX2 PE=1 SV=5	HUMAN	2
133	5.15	15.62	8.2	RRRRRsp|Q8WZ42|TITIN_HUMAN	REVERSED Titin OS=Homo sapiens GN=TTN PE=1 SV=2	HUMAN	3
134	4.6	4.73	14.9	sp|Q08380|LG3BP_HUMAN	Galectin-3-binding protein OS=Homo sapiens GN=LGALS3BP PE=1 SV=1	HUMAN	1
135	4.35	4.35	32	sp|P02775|CXCL7_HUMAN	Platelet basic protein OS=Homo sapiens GN=PPBP PE=1 SV=3	HUMAN	2
136	4.34	4.39	12	sp|O75882|ATRN_HUMAN	Attractin OS=Homo sapiens GN=ATRN PE=1 SV=2	HUMAN	1
137	4.24	4.24	49.5	sp|P05109|S10A8_HUMAN	Protein S100-A8 OS=Homo sapiens GN=S100A8 PE=1 SV=1	HUMAN	3
138	4.19	4.21	11.3	sp|P16070|CD44_HUMAN	CD44 antigen OS=Homo sapiens GN=CD44 PE=1 SV=2	HUMAN	2
139	4.11	4.11	27.7	sp|P61769|B2MG_HUMAN	Beta-2-microglobulin OS=Homo sapiens GN=B2M PE=1 SV=1	HUMAN	2
140	4.07	4.07	23.9	sp|P04278|SHBG_HUMAN	Sex hormone-binding globulin OS=Homo sapiens GN=SHBG PE=1 SV=2	HUMAN	2
141	4.02	12.08	27.6	sp|P01857|IGHG1_HUMAN	Ig gamma-1 chain C region OS=Homo sapiens GN=IGHG1 PE=1 SV=1	HUMAN	6
142	4.01	4.01	34.3	sp|P01842|LAC_HUMAN	Ig lambda chain C regions OS=Homo sapiens GN=IGLC1 PE=1 SV=1	HUMAN	2
143	4	4.13	10.6	sp|P33151|CADH5_HUMAN	Cadherin-5 OS=Homo sapiens GN=CDH5 PE=1 SV=4	HUMAN	2
144	4	4	15.6	sp|P55058|PLTP_HUMAN	Phospholipid transfer protein OS=Homo sapiens GN=PLTP PE=1 SV=1	HUMAN	2
145	4	4	33.6	sp|P07737|PROF1_HUMAN	Profilin-1 OS=Homo sapiens GN=PFN1 PE=1 SV=2	HUMAN	2
146	4	4	4.3	sp|Q6UXB8|PI16_HUMAN	Peptidase inhibitor 16 OS=Homo sapiens GN=PI16 PE=1 SV=1	HUMAN	2
147	3.78	6.2	27.4	sp|P35908|K22E_HUMAN	Keratin, type II cytoskeletal 2 epidermal OS=Homo sapiens GN=KRT2 PE=1 SV=1	HUMAN	3
148	3.4	3.4	70.5	sp|P62328|TYB4_HUMAN	Thymosin beta-4 OS=Homo sapiens GN=TMSB4X PE=1 SV=2	HUMAN	2
149	3.32	3.32	31.5	sp|P67936|TPM4_HUMAN	Tropomyosin alpha-4 chain OS=Homo sapiens GN=TPM4 PE=1 SV=3	HUMAN	2
150	3.23	3.23	3	RRRRRsp|Q4KMG0|CDON_HUMAN	REVERSED Cell adhesion molecule-related/down-regulated by oncogenes OS=Homo sapiens G	HUMAN	1
151	3.02	4.87	37.6	sp|P02776|PLF4_HUMAN	Platelet factor 4 OS=Homo sapiens GN=PF4 PE=1 SV=2	HUMAN	1
152	2.95	8.86	7.5	sp|Q8WZ42|TITIN_HUMAN	Titin OS=Homo sapiens GN=TTN PE=1 SV=2	HUMAN	0
153	2.95	3.07	6.5	sp|Q8TCU6|PREX1_HUMAN	Phosphatidylinositol 3,4,5-trisphosphate-dependent Rac exchanger 1 protein OS=Homo sapien	HUMAN	1
154	2.87	2.88	20.2	sp|P48740|MASP1_HUMAN	Complement-activating component of Ra-reactive factor OS=Homo sapiens GN=MASP1 PE=1 S	HUMAN	1
155	2.83	3.41	6	RRRRRsp|Q38SD2|LRRK1_HUMAN	REVERSED Leucine-rich repeat serine/threonine-protein kinase 1 OS=Homo sapiens GN=LRRK1	HUMAN	1
156	2.82	2.83	7.3	sp|P09172|DOPO_HUMAN	Dopamine beta-hydroxylase OS=Homo sapiens GN=DBH PE=1 SV=3	HUMAN	1
157	2.73	2.74	18.2	sp|Q15582|BGH3_HUMAN	Transforming growth factor-beta-induced protein ig-h3 OS=Homo sapiens GN=TGFBI PE=1 SV=	HUMAN	1
158	2.64	3.15	8.6	sp|P07359|GP1BA_HUMAN	Platelet glycoprotein Ib alpha chain OS=Homo sapiens GN=GP1BA PE=1 SV=1	HUMAN	1
159	2.63	2.7	4.2	RRRRRsp|P14314|GLU2B_HUMAN	REVERSED Glucosidase 2 subunit beta OS=Homo sapiens GN=PRKCSH PE=1 SV=2	HUMAN	1
160	2.59	2.59	9.9	sp|P20851|C4BPB_HUMAN	C4b-binding protein beta chain OS=Homo sapiens GN=C4BPB PE=1 SV=1	HUMAN	1
161	2.57	2.7	7.6	sp|P13591|NCAM1_HUMAN	Neural cell adhesion molecule 1 OS=Homo sapiens GN=NCAM1 PE=1 SV=3	HUMAN	1
162	2.55	2.55	10.7	sp|Q13790|APOF_HUMAN	Apolipoprotein F OS=Homo sapiens GN=APOF PE=1 SV=1	HUMAN	1
163	2.5	8.88	27.6	sp|P01859|IGHG2_HUMAN	Ig gamma-2 chain C region OS=Homo sapiens GN=IGHG2 PE=1 SV=2	HUMAN	4
164	2.48	4.12	9.4	RRRRRsp|Q5THJ4|VP13D_HUMAN	REVERSED Vacuolar protein sorting-associated protein 13D OS=Homo sapiens GN=VPS13D PE=	HUMAN	1
165	2.48	2.49	4.6	sp|Q76LX8|ATS13_HUMAN	A disintegrin and metalloproteinase with thrombospondin motifs 13 OS=Homo sapiens GN=AD	HUMAN	1
166	2.48	2.48	8.3	sp|P01344|IGF2_HUMAN	Insulin-like growth factor II OS=Homo sapiens GN=IGF2 PE=1 SV=1	HUMAN	1
167	2.45	3.85	8.4	RRRRRsp|Q6ZRR7|LRRC9_HUMAN	REVERSED Leucine-rich repeat-containing protein 9 OS=Homo sapiens GN=LRRC9 PE=2 SV=2	HUMAN	1
168	2.41	2.41	29.7	sp|P61626|LYSC_HUMAN	Lysozyme C OS=Homo sapiens GN=LYZ PE=1 SV=1	HUMAN	1
169	2.4	3.51	8.9	RRRRRsp|Q96L91|EP400_HUMAN	REVERSED E1A-binding protein p400 OS=Homo sapiens GN=EP400 PE=1 SV=3	HUMAN	2
170	2.28	2.43	21.2	sp|P63104|1433Z_HUMAN	14-3-3 protein zeta/delta OS=Homo sapiens GN=YWHAZ PE=1 SV=1	HUMAN	1
171	2.26	4.07	9.8	RRRRRsp|Q8WXX0|DYH7_HUMAN	REVERSED Dynein heavy chain 7, axonemal OS=Homo sapiens GN=DNAH7 PE=1 SV=1	HUMAN	2
172	2.19	4.09	8.9	RRRRRsp|Q13459|MYO9B_HUMAN	REVERSED Myosin-IXb OS=Homo sapiens GN=MYO9B PE=1 SV=2	HUMAN	2
173	2.17	3.56	9.1	RRRRRsp|Q9NZQ3|SPN90_HUMAN	REVERSED SH3 adapter protein SPIN90 OS=Homo sapiens GN=NCKIPSD PE=1 SV=1	HUMAN	2
174	2.17	2.72	14.9	sp|Q9NR77|PXMP2_HUMAN	Peroxisomal membrane protein 2 OS=Homo sapiens GN=PXMP2 PE=1 SV=3	HUMAN	1
175	2.15	2.18	7.9	sp|Q9NPH3|IL1AP_HUMAN	Interleukin-1 receptor accessory protein OS=Homo sapiens GN=IL1RAP PE=1 SV=2	HUMAN	1
176	2.15	2.18	15.1	sp|Q9NZT1|CALL5_HUMAN	Calmodulin-like protein 5 OS=Homo sapiens GN=CALML5 PE=1 SV=2	HUMAN	1
177	2.14	3.1	6.5	RRRRRsp|Q8N3K9|CMYA5_HUMAN	REVERSED Cardiomyopathy-associated protein 5 OS=Homo sapiens GN=CMYA5 PE=1 SV=3	HUMAN	1
178	2.14	2.15	6.3	sp|Q5FWF5|ESCO1_HUMAN	N-acetyltransferase ESCO1 OS=Homo sapiens GN=ESCO1 PE=1 SV=3	HUMAN	1

179	2.12	2.35	4.2	RRRRRsp|O94915|FRYL_HUMAN	REVERSED Protein furry homolog-like OS=Homo sapiens GN=FRYL PE=1 SV=2	HUMAN	1
180	2.11	3.85	19.4	RRRRRsp|P04899|GNAI2_HUMAN	REVERSED Guanine nucleotide-binding protein G(i), alpha-2 subunit OS=Homo sapiens GN=GN	HUMAN	2
181	2.11	2.49	17.5	sp|P0C875|U638B_HUMAN	UPF0638 protein B OS=Homo sapiens PE=2 SV=1	HUMAN	1
182	2.11	2.11	8.1	sp|Q96KN2|CNDP1_HUMAN	Beta-Ala-His dipeptidase OS=Homo sapiens GN=CNDP1 PE=1 SV=3	HUMAN	1
183	2.11	2.11	12.1	sp|P08294|SODE_HUMAN	Extracellular superoxide dismutase [Cu-Zn] OS=Homo sapiens GN=SOD3 PE=1 SV=2	HUMAN	1
184	2.1	2.39	5.6	sp|P22105|TENX_HUMAN	Tenascin-X OS=Homo sapiens GN=TNXB PE=1 SV=2	HUMAN	1
185	2.1	2.1	9.6	RRRRRsp|Q6P1J9|CDC73_HUMAN	REVERSED Parafibromin OS=Homo sapiens GN=CDC73 PE=1 SV=1	HUMAN	1
186	2.1	2.1	22.5	sp|P01040|CYTA_HUMAN	Cystatin-A OS=Homo sapiens GN=CSTA PE=1 SV=1	HUMAN	1
187	2.09	13.78	16.7	cont|000108	gi|3024050|sp|O02668|ITIH2_PIG Inter-alpha-trypsin inhibitor heavy chain H2 precursor (ITI h	Sus scrofa	15
188	2.08	2.08	6.1	sp|P04180|LCAT_HUMAN	Phosphatidylcholine-sterol acyltransferase OS=Homo sapiens GN=LCAT PE=1 SV=1	HUMAN	1
189	2.07	2.07	3.5	RRRRRsp|Q6V1P9|PCD23_HUMAN	REVERSED Protocadherin-23 OS=Homo sapiens GN=DCHS2 PE=2 SV=1	HUMAN	1
190	2.06	2.79	9.3	sp|Q86UX7|URP2_HUMAN	Fermitin family homolog 3 OS=Homo sapiens GN=FERMT3 PE=1 SV=1	HUMAN	1
191	2.06	2.48	6.5	sp|Q02763|TIE2_HUMAN	Angiopoietin-1 receptor OS=Homo sapiens GN=TEK PE=1 SV=2	HUMAN	1
192	2.06	2.25	14	sp|P13796|PLSL_HUMAN	Plastin-2 OS=Homo sapiens GN=LCP1 PE=1 SV=5	HUMAN	1
193	2.06	2.15	5.5	sp|Q86VB7|C163A_HUMAN	Scavenger receptor cysteine-rich type 1 protein M130 OS=Homo sapiens GN=CD163 PE=1 SV=1	HUMAN	1
194	2.05	3.3	4.5	sp|Q7Z6Z7|HUWE1_HUMAN	E3 ubiquitin-protein ligase HUWE1 OS=Homo sapiens GN=HUWE1 PE=1 SV=3	HUMAN	1
195	2.04	2.04	10.3	sp|Q53EZ4|CEP55_HUMAN	Centrosomal protein of 55 kDa OS=Homo sapiens GN=CEP55 PE=1 SV=2	HUMAN	1
196	2.04	2.04	6.8	sp|P04040|CATA_HUMAN	Catalase OS=Homo sapiens GN=CAT PE=1 SV=3	HUMAN	1
197	2.03	2.03	20.9	sp|O43447|PPIH_HUMAN	Peptidyl-prolyl cis-trans isomerase H OS=Homo sapiens GN=PPIH PE=1 SV=1	HUMAN	1
198	2.02	2.23	14.1	sp|Q9UGM5|FETUB_HUMAN	Fetuin-B OS=Homo sapiens GN=FETUB PE=1 SV=2	HUMAN	1
199	2.02	2.04	7.5	RRRRRsp|Q9UH65|SWP70_HUMAN	REVERSED Switch-associated protein 70 OS=Homo sapiens GN=SWAP70 PE=1 SV=1	HUMAN	1
200	2.02	2.04	6.4	RRRRRsp|Q14746|COG2_HUMAN	REVERSED Conserved oligomeric Golgi complex subunit 2 OS=Homo sapiens GN=COG2 PE=1 SV	HUMAN	1
201	2.02	2.03	3.6	sp|Q5VV43|K0319_HUMAN	Uncharacterized protein KIAA0319 OS=Homo sapiens GN=KIAA0319 PE=1 SV=1	HUMAN	1
202	2.01	2.36	4.1	sp|Q92547|TOPB1_HUMAN	DNA topoisomerase 2-binding protein 1 OS=Homo sapiens GN=TOPBP1 PE=1 SV=2	HUMAN	1
203	2.01	2.2	14.7	RRRRRsp|Q9H943|CJ068_HUMAN	REVERSED Uncharacterized protein C10orf68 OS=Homo sapiens GN=C10orf68 PE=2 SV=1	HUMAN	1
204	2.01	2.03	6.5	sp|Q8NEM2|SHCBP_HUMAN	SHC SH2 domain-binding protein 1 OS=Homo sapiens GN=SHCBP1 PE=1 SV=2	HUMAN	1
205	2.01	2.02	7.7	sp|P43121|MUC18_HUMAN	Cell surface glycoprotein MUC18 OS=Homo sapiens GN=MCAM PE=1 SV=2	HUMAN	1
206	2.01	2.01	14.6	sp|Q9Y600|CSAD_HUMAN	Cysteine sulfinic acid decarboxylase OS=Homo sapiens GN=CSAD PE=1 SV=2	HUMAN	1
207	2.01	2.01	23.5	sp|P01777|HV316_HUMAN	Ig heavy chain V-III region TEI OS=Homo sapiens PE=1 SV=1	HUMAN	1
208	2	6.38	27.8	sp|P36980|FHR2_HUMAN	Complement factor H-related protein 2 OS=Homo sapiens GN=CFHR2 PE=1 SV=1	HUMAN	7
209	2	2.38	8.7	sp|Q96EP9|NTCP4_HUMAN	Sodium/bile acid cotransporter 4 OS=Homo sapiens GN=SLC10A4 PE=2 SV=2	HUMAN	1
210	2	2.15	9.8	sp|Q8N5M9|JAGN1_HUMAN	Protein jagunal homolog 1 OS=Homo sapiens GN=JAGN1 PE=2 SV=1	HUMAN	1
211	2	2.05	7.9	sp|Q6EMK4|VASN_HUMAN	Vasorin OS=Homo sapiens GN=VASN PE=1 SV=1	HUMAN	1
212	2	2.01	12.4	sp|P10599|THIO_HUMAN	Thioredoxin OS=Homo sapiens GN=TXN PE=1 SV=3	HUMAN	1
213	2	2	9.5	sp|P22891|PROZ_HUMAN	Vitamin K-dependent protein Z OS=Homo sapiens GN=PROZ PE=1 SV=2	HUMAN	1
214	2	2	24.5	sp|P03950|ANGI_HUMAN	Angiogenin OS=Homo sapiens GN=ANG PE=1 SV=1	HUMAN	1
215	2	2	5.6	sp|Q9Y5Y7|LYVE1_HUMAN	Lymphatic vessel endothelial hyaluronic acid receptor 1 OS=Homo sapiens GN=LYVE1 PE=1 SV=	HUMAN	1
216	2	2	4.3	sp|P12955|PEPD_HUMAN	Xaa-Pro dipeptidase OS=Homo sapiens GN=PEPD PE=1 SV=3	HUMAN	1
217	2	2	5.2	sp|P04001|OPSG_HUMAN	Green-sensitive opsin OS=Homo sapiens GN=OPN1MW PE=1 SV=1	HUMAN	1
218	2	2	18.9	sp|P02735|SAA_HUMAN	Serum amyloid A protein OS=Homo sapiens GN=SAA1 PE=1 SV=2	HUMAN	1
219	2	2	16.4	sp|P01034|CYTC_HUMAN	Cystatin-C OS=Homo sapiens GN=CST3 PE=1 SV=1	HUMAN	1
220	2	2	2.7	sp|P02741|CRP_HUMAN	C-reactive protein OS=Homo sapiens GN=CRP PE=1 SV=1	HUMAN	1

Supplementary table 1 (8plex experiment)

N	Unused	Total	% Cov	Accession #	Name	Species	Peptides(95%)
2	234.19	234.22	69.3	P01024|CO3_HUMAN	Complement C3 precursor [Contains: Complement C3 beta chain; Complement C3 alpha chain; C3a anaphylatoxin; Complement C3b alp	HUMAN	215
1	268.04	268.04	55.4	P04114|APOB_HUMAN	Apolipoprotein B-100 precursor (Apo B-100) [Contains: Apolipoprotein B-48 (Apo B-48)] - Homo sapiens (Human)	HUMAN	162
3	145.98	145.99	58.5	P01023|A2MG_HUMAN	Alpha-2-macroglobulin precursor (Alpha-2-M) - Homo sapiens (Human)	HUMAN	146
5	107.08	107.11	62	P02671|FIBA_HUMAN	Fibrinogen alpha chain precursor [Contains: Fibrinopeptide A] - Homo sapiens (Human)	HUMAN	137
4	133.84	135.22	50	P0C0L5|CO4B_HUMAN	Complement C4-B precursor (Basic complement C4) [Contains: Complement C4 beta chain; Complement C4-B alpha chain; C4a anaphyl	HUMAN	98
9	62.9	62.9	90.3	P02647|APOA1_HUMAN	Apolipoprotein A-I precursor (Apo-AI) (ApoA-I) [Contains: Apolipoprotein A-I(1-242)] - Homo sapiens (Human)	HUMAN	98
6	96.86	96.86	66.4	P02675|FIBB_HUMAN	Fibrinogen beta chain precursor [Contains: Fibrinopeptide B] - Homo sapiens (Human)	HUMAN	96
8	76.38	76.44	73.5	P02679|FIBG_HUMAN	Fibrinogen gamma chain precursor - Homo sapiens (Human)	HUMAN	90
7	89.44	90	36.3	P02751|FINC_HUMAN	Fibronectin precursor (FN) (Cold-insoluble globulin) (CIG) - Homo sapiens (Human)	HUMAN	66
12	55.53	55.64	43.3	P00450|CERU_HUMAN	Ceruloplasmin precursor (EC 1.16.3.1) (Ferroxidase) - Homo sapiens (Human)	HUMAN	59
11	60.64	60.64	52.6	P02790|HEMO_HUMAN	Hemopexin precursor (Beta-1B-glycoprotein) - Homo sapiens (Human)	HUMAN	55
10	61.72	61.72	81.6	P06727|APOA4_HUMAN	Apolipoprotein A-IV precursor (Apo-AIV) (ApoA-IV) - Homo sapiens (Human)	HUMAN	43
13	48.47	49.1	36	P00751|CFAB_HUMAN	Complement factor B precursor (EC 3.4.21.47) (C3/C5 convertase) (Properdin factor B) (Glycine-rich beta glycoprotein) (GBG) (PBF2) [C	HUMAN	36
14	47.06	47.19	44	Q14624|ITIH4_HUMAN	Inter-alpha-trypsin inhibitor heavy chain H4 precursor (ITI heavy chain H4) (Inter-alpha-inhibitor heavy chain 4) (Inter-alpha-trypsin inhi	HUMAN	34
17	44.16	44.17	34.1	P19823|ITIH2_HUMAN	Inter-alpha-trypsin inhibitor heavy chain H2 precursor (ITI heavy chain H2) (Inter-alpha-inhibitor heavy chain 2) (Inter-alpha-trypsin inhi	HUMAN	34
22	30.1	30.13	42.6	P01011|AACT_HUMAN	Alpha-1-antichymotrypsin precursor (ACT) [Contains: Alpha-1-antichymotrypsin His-Pro-less] - Homo sapiens (Human)	HUMAN	34
15	45.78	45.79	45.5	P01008|ANT3_HUMAN	Antithrombin-III precursor (ATIII) - Homo sapiens (Human)	HUMAN	32
16	45.42	45.45	28.9	P08603|CFAH_HUMAN	Complement factor H precursor (H factor 1) - Homo sapiens (Human)	HUMAN	29
24	29.61	29.65	46.3	P02763|A1AG1_HUMAN	Alpha-1-acid glycoprotein 1 precursor (AGP 1) (Orosomucoid-1) (OMD 1) - Homo sapiens (Human)	HUMAN	27
133	2.61	26.58	29.8	P20742|PZP_HUMAN	Pregnancy zone protein precursor - Homo sapiens (Human)	HUMAN	27
19	33.75	34.48	32.6	P19827|ITIH1_HUMAN	Inter-alpha-trypsin inhibitor heavy chain H1 precursor (ITI heavy chain H1) (Inter-alpha-inhibitor heavy chain 1) (Inter-alpha-trypsin inhi	HUMAN	24
27	27.61	27.63	32.6	P01042|KNG1_HUMAN	Kininogen-1 precursor (Alpha-2-thiol proteinase inhibitor) [Contains: Kininogen-1 heavy chain; Bradykinin (Kallidin I); Lysyl-bradykinin (K	HUMAN	24
28	27.34	27.34	40.6	P05155|IC1_HUMAN	Plasma protease C1 inhibitor precursor (C1 Inh) (C1Inh) (C1 esterase inhibitor) (C1-inhibiting factor) - Homo sapiens (Human)	HUMAN	24
18	40.09	40.31	30.7	P01031|CO5_HUMAN	Complement C5 precursor [Contains: Complement C5 beta chain; Complement C5 alpha chain; C5a anaphylatoxin; Complement C5 alph	HUMAN	23
21	30.28	30.29	52.1	P05546|HEP2_HUMAN	Heparin cofactor 2 precursor (Heparin cofactor II) (HC-II) (Protease inhibitor leuserpin 2) (HLS2) - Homo sapiens (Human)	HUMAN	20
23	29.75	30.16	36.7	P02774|VTDB_HUMAN	Vitamin D-binding protein precursor (DBP) (Group-specific component) (Gc-globulin) (VDB) - Homo sapiens (Human)	HUMAN	20
39	21.5	21.64	42	P02765|FETUA_HUMAN	Alpha-2-HS-glycoprotein precursor (Fetuin-A) (Alpha-2-Z-globulin) (Ba-alpha-2-glycoprotein) [Contains: Alpha-2-HS-glycoprotein chain A	HUMAN	20
77	9.05	14.62	35.8	P19652|A1AG2_HUMAN	Alpha-1-acid glycoprotein 2 precursor (AGP 2) (Orosomucoid-2) (OMD 2) - Homo sapiens (Human)	HUMAN	20
20	32.97	33.34	39.9	P06396|GELS_HUMAN	Gelsolin precursor (Actin-depolymerizing factor) (ADF) (Brevin) (AGEL) - Homo sapiens (Human)	HUMAN	18
26	28.91	28.91	65	P02649|APOE_HUMAN	Apolipoprotein E precursor (Apo-E) - Homo sapiens (Human)	HUMAN	17
29	25.35	25.36	41.2	P10909|CLUS_HUMAN	Clusterin precursor (Complement-associated protein SP-40,40) (Complement cytolysis inhibitor) (CLI) (NA1/NA2) (Apolipoprotein J) (Ap	HUMAN	17
36	21.73	21.76	32.7	P04217|A1BG_HUMAN	Alpha-1B-glycoprotein precursor (Alpha-1-B glycoprotein) - Homo sapiens (Human)	HUMAN	17
25	29.55	29.67	31.5	P00747|PLMN_HUMAN	Plasminogen precursor (EC 3.4.21.7) [Contains: Plasmin heavy chain A; Activation peptide; Angiostatin; Plasmin heavy chain A, short for	HUMAN	16
33	23.3	23.59	40.2	P00734|THRB_HUMAN	Prothrombin precursor (EC 3.4.21.5) (Coagulation factor II) [Contains: Activation peptide fragment 1; Activation peptide fragment 2; Th	HUMAN	16
34	23.07	23.11	29.1	P04196|HRG_HUMAN	Histidine-rich glycoprotein precursor (Histidine-proline-rich glycoprotein) (HPRG) - Homo sapiens (Human)	HUMAN	16
38	21.54	21.88	46.1	P25311|ZA2G_HUMAN	Zinc-alpha-2-glycoprotein precursor (Zn-alpha-2-glycoprotein) (Zn-alpha-2-GP) - Homo sapiens (Human)	HUMAN	16
35	22.26	23.78	25.2	P43652|AFAM_HUMAN	Afamin precursor (Alpha-albumin) (Alpha-Alb) - Homo sapiens (Human)	HUMAN	15
46	16.89	16.89	67	P02652|APOA2_HUMAN	Apolipoprotein A-II precursor (Apo-AII) (ApoA-II) [Contains: Apolipoprotein A-II(1-76)] - Homo sapiens (Human)	HUMAN	15
30	24.45	24.47	29.7	P08697|A2AP_HUMAN	Alpha-2-antiplasmin precursor (Alpha-2-plasmin inhibitor) (Alpha-2-PI) (Alpha-2-AP) - Homo sapiens (Human)	HUMAN	14
37	21.63	21.67	36.8	P04004|VTNC_HUMAN	Vitronectin precursor (Serum-spreading factor) (S-protein) (V75) [Contains: Vitronectin V65 subunit; Vitronectin V10 subunit; Somatom	HUMAN	14
32	23.93	23.95	44	P01009|A1AT_HUMAN	Alpha-1-antitrypsin precursor (Alpha-1 protease inhibitor) (Alpha-1-antiproteinase) - Homo sapiens (Human)	HUMAN	13
41	19.74	19.77	28.4	P07358|CO8B_HUMAN	Complement component C8 beta chain precursor (Complement component 8 subunit beta) - Homo sapiens (Human)	HUMAN	13
56	13.33	14.14	42.2	P02743|SAMP_HUMAN	Serum amyloid P-component precursor (SAP) (9.5S alpha-1-glycoprotein) [Contains: Serum amyloid P-component(1-203)] - Homo sapie	HUMAN	13
31	23.93	23.95	23.2	P13671|CO6_HUMAN	Complement component C6 precursor - Homo sapiens (Human)	HUMAN	12
40	19.94	19.95	33.7	P00738|HPT_HUMAN	Haptoglobin precursor [Contains: Haptoglobin alpha chain; Haptoglobin beta chain] - Homo sapiens (Human)	HUMAN	11
42	18.85	18.86	36.9	P02750|A2GL_HUMAN	Leucine-rich alpha-2-glycoprotein precursor (LRG) - Homo sapiens (Human)	HUMAN	11
44	18.62	18.83	30.4	P03952|KLKB1_HUMAN	Plasma kallikrein precursor (EC 3.4.21.34) (Plasma prekallikrein) (Kininogenin) (Fletcher factor) [Contains: Plasma kallikrein heavy chain	HUMAN	11
45	17.11	17.13	21.7	P09871|C1S_HUMAN	Complement C1s subcomponent precursor (EC 3.4.21.42) (C1 esterase) [Contains: Complement C1s subcomponent heavy chain; Compl	HUMAN	10
47	15.34	15.42	36.1	P51884|LUM_HUMAN	Lumican precursor (Keratan sulfate proteoglycan lumican) (KSPG lumican) - Homo sapiens (Human)	HUMAN	10
53	13.69	13.73	32.8	P02749|APOH_HUMAN	Beta-2-glycoprotein 1 precursor (Beta-2-glycoprotein I) (Apolipoprotein H) (Apo-H) (B2GPI) (Beta(2)GPI) (Activated protein C-binding pr	HUMAN	10
91	6.01	6.01	37.4	P02656|APOC3_HUMAN	Apolipoprotein C-III precursor (Apo-CIII) (ApoC-III) - Homo sapiens (Human)	HUMAN	10
51	14.13	15.56	29.9	P06681|CO2_HUMAN	Complement C2 precursor (EC 3.4.21.43) (C3/C5 convertase) [Contains: Complement C2b fragment; Complement C2a fragment] - Homo	HUMAN	9
81	8.09	8.14	58	P02747|C1QC_HUMAN	Complement C1q subcomponent subunit C precursor - Homo sapiens (Human)	HUMAN	9
43	18.84	18.84	34.4	P01019|ANGT_HUMAN	Angiotensinogen precursor [Contains: Angiotensin-1 (Angiotensin I) (Ang I); Angiotensin-2 (Angiotensin II) (Ang II); Angiotensin-3 (Angio	HUMAN	8
52	14.02	14.02	66.3	P02654|APOC1_HUMAN	Apolipoprotein C-I precursor (Apo-CI) (ApoC-I) - Homo sapiens (Human)	HUMAN	8
60	12.5	12.5	55.1	P68871|HBB_HUMAN	Hemoglobin subunit beta (Hemoglobin beta chain) (Beta-globin) - Homo sapiens (Human)	HUMAN	8
73	9.4	9.4	26.7	P02760|AMBP_HUMAN	AMBP protein precursor [Contains: Alpha-1-microglobulin (Protein HC) (Complex-forming glycoprotein heterogeneous in charge) (Alph	HUMAN	8
48	15.19	16.55	24.5	Q06033|ITIH3_HUMAN	Inter-alpha-trypsin inhibitor heavy chain H3 precursor (ITI heavy chain H3) (Inter-alpha-inhibitor heavy chain 3) (Serum-derived hyaluro	HUMAN	7
50	14.59	14.74	39	P36955|PEDF_HUMAN	Pigment epithelium-derived factor precursor (PEDF) (Serpin-F1) (EPC-1) - Homo sapiens (Human)	HUMAN	7
55	13.51	13.51	29.2	P02748|CO9_HUMAN	Complement component C9 precursor [Contains: Complement component C9a; Complement component C9b] - Homo sapiens (Human)	HUMAN	7
58	12.99	13.06	24.5	P02787|TRFE_HUMAN	Serotransferrin precursor (Transferrin) (Siderophilin) (Beta-1-metal-binding globulin) - Homo sapiens (Human)	HUMAN	7
72	9.53	9.53	39.3	P02753|RETBP_HUMAN	Plasma retinol-binding protein precursor (PRBP) (RBP) [Contains: Plasma retinol-binding protein(1-182); Plasma retinol-binding protein(	HUMAN	7
49	15.07	15.09	31.7	P35858|ALS_HUMAN	Insulin-like growth factor-binding protein complex acid labile chain precursor (ALS) - Homo sapiens (Human)	HUMAN	6
54	13.65	13.65	24.8	P27169|PON1_HUMAN	Serum paraoxonase/arylesterase 1 (EC 3.1.1.2) (EC 3.1.8.1) (PON 1) (Serum aryldialkylphosphatase 1) (A-esterase 1) (Aromatic esterase	HUMAN	6
57	13.2	13.66	32.8	P29622|KAIN_HUMAN	Kallistatin precursor (Serpin A4) (Kallikrein inhibitor) (Protease inhibitor 4) - Homo sapiens (Human)	HUMAN	6
59	12.95	12.98	19.2	P07357|CO8A_HUMAN	Complement component C8 alpha chain precursor (Complement component 8 subunit alpha) - Homo sapiens (Human)	HUMAN	6
61	12.34	12.35	25.1	P13645|K1C10_HUMAN	Keratin, type I cytoskeletal 10 (Cytokeratin-10) (CK-10) (Keratin-10) (K10) - Homo sapiens (Human)	HUMAN	6
62	12.17	12.17	38.4	P63261|ACTG_HUMAN	Actin, cytoplasmic 2 (Gamma-actin) - Homo sapiens (Human)	HUMAN	6
63	11.66	13.2	40.1	P04264|K2C1_HUMAN	Keratin, type II cytoskeletal 1 (Cytokeratin-1) (CK-1) (Keratin-1) (K1) (67 kDa cytokeratin) (Hair alpha protein) - Homo sapiens (Human)	HUMAN	6
65	10.96	10.97	21.4	P01871|MUC_HUMAN	Ig mu chain C region - Homo sapiens (Human)	HUMAN	6
66	10.95	12.97	27.5	P05543|THBG_HUMAN	Thyroxine-binding globulin precursor (T4-binding globulin) (Serpin A7) - Homo sapiens (Human)	HUMAN	6
79	8.21	8.21	59.4	P02655|APOC2_HUMAN	Apolipoprotein C-II precursor (Apo-CII) (ApoC-II) - Homo sapiens (Human)	HUMAN	6
64	10.96	12.21	25.1	P02768|ALBU_HUMAN	Serum albumin precursor - Homo sapiens (Human)	HUMAN	5
68	10.45	10.45	18.1	P80108|PHLD1_HUMAN	Phosphatidylinositol-glycan-specific phospholipase D 1 precursor (EC 3.1.4.50) (PI-G PLD) (Glycoprotein phospholipase D) (Glycosyl-pho	HUMAN	5
69	10.15	10.17	24	P22792|CPN2_HUMAN	Carboxypeptidase N subunit 2 precursor (Carboxypeptidase N polypeptide 2) (Carboxypeptidase N 83 kDa chain) (Carboxypeptidase N r	HUMAN	5
74	9.17	9.17	43.4	P05090|APOD_HUMAN	Apolipoprotein D precursor (Apo-D) (ApoD) - Homo sapiens (Human)	HUMAN	5
75	9.12	9.13	22.3	P10643|CO7_HUMAN	Complement component C7 precursor - Homo sapiens (Human)	HUMAN	5
78	8.75	8.75	40.8	P35542|SAA4_HUMAN	Serum amyloid A-4 protein precursor (Constitutively expressed serum amyloid A protein) (C-SAA) - Homo sapiens (Human)	HUMAN	5
82	8.06	8.08	23.4	P07225|PROS_HUMAN	Vitamin K-dependent protein S precursor - Homo sapiens (Human)	HUMAN	5
67	10.82	10.82	25.9	P05156|CFAI_HUMAN	Complement factor I precursor (EC 3.4.21.45) (C3B/C4B inactivator) [Contains: Complement factor I heavy chain; Complement factor I li	HUMAN	4
71	9.61	9.75	31.9	O14791|APOL1_HUMAN	Apolipoprotein-L1 precursor (Apolipoprotein L-I) (Apolipoprotein L) (ApoL-I) (Apo-L) (ApoL) - Homo sapiens (Human)	HUMAN	4
76	9.07	9.09	21	P00736|C1R_HUMAN	Complement C1r subcomponent precursor (EC 3.4.21.41) (Complement component 1, r subcomponent) [Contains: Complement C1r su	HUMAN	4
80	8.13	8.39	21.8	Q96IY4|CBPB2_HUMAN	Carboxypeptidase B2 precursor (EC 3.4.17.20) (Carboxypeptidase U) (CPU) (Thrombin-activable fibrinolysis inhibitor) (TAFI) (Plasma car	HUMAN	4
83	7.74	7.75	55	P07360|CO8G_HUMAN	Complement component C8 gamma chain precursor - Homo sapiens (Human)	HUMAN	4
88	6.29	6.29	36.6	P69905|HBA_HUMAN	Hemoglobin subunit alpha (Hemoglobin alpha chain) (Alpha-globin) - Homo sapiens (Human)	HUMAN	4
70	10.04	18.09	19	Q8WZ42|TITIN_HUMAN	Titin (EC 2.7.11.1) (Connectin) (Rhabdomyosarcoma antigen MU-RMS-40.14) - Homo sapiens (Human)	HUMAN	3
85	6.8	6.8	15.5	P43251|BTD_HUMAN	Biotinidase precursor (EC 3.5.1.12) - Homo sapiens (Human)	HUMAN	3
86	6.75	8.34	12.7	P12259|FA5_HUMAN	Coagulation factor V precursor (Activated protein C cofactor) [Contains: Coagulation factor V heavy chain; Coagulation factor V light cha	HUMAN	3
87	6.32	6.67	38.8	P02766|TTHY_HUMAN	Transthyretin precursor (Prealbumin) (TBPA) (TTR) (ATTR) - Homo sapiens (Human)	HUMAN	3
89	6.12	6.15	24	P08185|CBG_HUMAN	Corticosteroid-binding globulin precursor (CBG) (Transcortin) (Serpin A6) - Homo sapiens (Human)	HUMAN	3
90	6.02	6.02	25.2	P22352|GPX3_HUMAN	Glutathione peroxidase 3 precursor (EC 1.11.1.9) (GSHPx-3) (GPx-3) (Plasma glutathione peroxidase) (GSHPx-P) (Extracellular glutathion	HUMAN	3
92	5.83	5.83	28.7	P02746|C1QB_HUMAN	Complement C1q subcomponent subunit B precursor - Homo sapiens (Human)	HUMAN	3
93	5.52	5.52	24.8	P32119|PRDX2_HUMAN	Peroxiredoxin-2 (EC 1.11.1.15) (Thioredoxin peroxidase 1) (Thioredoxin-dependent peroxide reductase 1) (Thiol-specific antioxidant pro	HUMAN	3
94	5.4	5.42	25.7	P05452|TETN_HUMAN	Tetranectin precursor (TN) (C-type lectin domain family 3 member B) (Plasminogen kringle 4-binding protein) - Homo sapiens (Human)	HUMAN	3
111	3.87	3.88	22	P02745|C1QA_HUMAN	Complement C1q subcomponent subunit A precursor - Homo sapiens (Human)	HUMAN	3
131	2.66	4.69	27.5	P35527|K1C9_HUMAN	Keratin, type I cytoskeletal 9 (Cytokeratin-9) (CK-9) (Keratin-9) (K9) - Homo sapiens (Human)	HUMAN	3
181	2.04	3.39	15.8	Q15746|MYLK_HUMAN	Myosin light chain kinase, smooth muscle (EC 2.7.11.18) (MLCK) (Telokin) (Kinase-related protein) (KRP) - Homo sapiens (Human)	HUMAN	3
95	5.25	5.32	21.2	P15169|CBPN_HUMAN	Carboxypeptidase N catalytic chain precursor (EC 3.4.17.3) (CPN) (Carboxypeptidase N polypeptide 1) (Carboxypeptidase N small subuni	HUMAN	2
96	4.89	4.9	14.4	P49908|SEPP1_HUMAN	Selenoprotein P precursor (SeP) - Homo sapiens (Human)	HUMAN	2
97	4.76	4.97	12.9	P05160|F13B_HUMAN	Coagulation factor XIII B chain precursor (Protein-glutamine gamma-glutamyltransferase B chain) (Transglutaminase B chain) (Fibrin-sta	HUMAN	2

98	4.61	4.61	19.3	Q16610|ECM1_HUMAN	Extracellular matrix protein 1 precursor (Secretory component p85) - Homo sapiens (Human)	HUMAN	2
99	4.57	5.33	23.5	P35580|MYH10_HUMAN	Myosin-10 (Myosin heavy chain 10) (Myosin heavy chain, nonmuscle IIb) (Nonmuscle myosin heavy chain IIb) (NMMHC II-b) (NMMHC-I	HUMAN	2
100	4.33	4.4	17.3	P04003|C4BP_HUMAN	C4b-binding protein alpha chain precursor (C4bp) (Proline-rich protein) (PRP) - Homo sapiens (Human)	HUMAN	2
102	4.27	4.76	14.5	P05154|IPSP_HUMAN	Plasma serine protease inhibitor precursor (PCI) (Protein C inhibitor) (Serpin A5) (Plasminogen activator inhibitor 3) (PAI-3) (PAI3) (Acro	HUMAN	2
103	4.26	4.34	28.7	P00915|CAH1_HUMAN	Carbonic anhydrase 1 (EC 4.2.1.1) (Carbonic anhydrase I) (Carbonate dehydratase I) (CA-I) - Homo sapiens (Human)	HUMAN	2
104	4.18	4.18	9.3	RRRRRQ9Y3M8|STA13_HU	REVERSED StAR-related lipid transfer protein 13 (StARD13) (START domain-containing protein 13) (46H23.2) (Deleted in liver cancer pro	HUMAN	2
105	4.15	4.16	20.7	O75636|FCN3_HUMAN	Ficolin-3 precursor (Collagen/fibrinogen domain-containing protein 3) (Collagen/fibrinogen domain-containing lectin 3 p35) (Hakata ant	HUMAN	2
106	4.07	4.1	13.1	P06276|CHLE_HUMAN	Cholinesterase precursor (EC 3.1.1.8) (Acylcholine acylhydrolase) (Choline esterase II) (Butyrylcholine esterase) (Pseudocholinesterase)	HUMAN	2
107	4.04	4.04	26.6	P26927|HGFL_HUMAN	Hepatocyte growth factor-like protein precursor (Macrophage stimulatory protein) (MSP) (Macrophage-stimulating protein) [Contains:	HUMAN	2
108	4.02	4.02	43.3	P55056|APOC4_HUMAN	Apolipoprotein C-IV precursor (Apo-CIV) (ApoC-IV) - Homo sapiens (Human)	HUMAN	2
109	4.01	4.02	8.5	O75882|ATRN_HUMAN	Attractin precursor (Mahogany homolog) (DPPT-L) - Homo sapiens (Human)	HUMAN	2
110	4	4.03	11.6	P04180|LCAT_HUMAN	Phosphatidylcholine-sterol acyltransferase precursor (EC 2.3.1.43) (Lecithin-cholesterol acyltransferase) (Phospholipid-cholesterol acylt	HUMAN	2
112	3.7	3.7	45.5	P62328|TYB4_HUMAN	Thymosin beta-4 (T beta 4) (Fx) [Contains: Hematopoietic system regulatory peptide (Seraspenide)] - Homo sapiens (Human)	HUMAN	2
113	3.64	3.64	9.8	P23142|FBLN1_HUMAN	Fibulin-1 precursor - Homo sapiens (Human)	HUMAN	2
114	3.62	4.2	15	Q04756|HGFA_HUMAN	Hepatocyte growth factor activator precursor (EC 3.4.21.-) (HGF activator) (HGFA) [Contains: Hepatocyte growth factor activator short c	HUMAN	2
115	3.59	3.59	25	O95445|APOM_HUMAN	Apolipoprotein M (Apo-M) (ApoM) (Protein G3a) - Homo sapiens (Human)	HUMAN	2
116	3.54	3.55	17.8	P00742|FA10_HUMAN	Coagulation factor X precursor (EC 3.4.21.6) (Stuart factor) (Stuart-Prower factor) [Contains: Factor X light chain; Factor X heavy chain;	HUMAN	2
156	2.18	5.64	21.4	RRRRRO15083|ERC2_HUMA	REVERSED ERC protein 2 - Homo sapiens (Human)	HUMAN	2
164	2.13	2.15	17.8	RRRRRQ4V328|GRAP1_HU	REVERSED GRIP1-associated protein 1 (GRASP-1) - Homo sapiens (Human)	HUMAN	2
176	2.07	3.47	9.4	P08519|APOA_HUMAN	Apolipoprotein(a) precursor (EC 3.4.21.-) (Apo(a)) (Lp(a)) - Homo sapiens (Human)	HUMAN	2
195	2.01	2.31	29.1	Q03591|FHR1_HUMAN	Complement factor H-related protein 1 precursor (FHR-1) (H factor-like protein 1) (H-factor-like 1) (H36) - Homo sapiens (Human)	HUMAN	2
299	1.39	2.77	9.9	P03951|FA11_HUMAN	Coagulation factor XI precursor (EC 3.4.21.27) (Plasma thromboplastin antecedent) (PTA) (FXI) [Contains: Coagulation factor XIa heavy c	HUMAN	2
84	6.96	12.95	18.6	RRRRRQ8WZ42|TITIN_HUM	REVERSED Titin (EC 2.7.11.1) (Connectin) (Rhabdomyosarcoma antigen MU-RMS-40.14) - Homo sapiens (Human)	HUMAN	1
117	3.42	4.16	13.9	Q9Y6V0|PCLO_HUMAN	Protein piccolo (Aczonin) - Homo sapiens (Human)	HUMAN	1
118	3.37	3.38	19.5	P00488|F13A_HUMAN	Coagulation factor XIII A chain precursor (EC 2.3.2.13) (Coagulation factor XIIIa) (Protein-glutamine gamma-glutamyltransferase A chain	HUMAN	1
119	3.32	4.35	18.8	RRRRRQ9NRC6|SPTN5_HU	REVERSED Spectrin beta chain, brain 4 (Spectrin, non-erythroid beta chain 4) (Beta-V spectrin) (BSPECV) - Homo sapiens (Human)	HUMAN	1
120	3.06	3.06	11.1	P07996|TSP1_HUMAN	Thrombospondin-1 precursor - Homo sapiens (Human)	HUMAN	1
122	2.92	3	11	P04275|VWF_HUMAN	von Willebrand factor precursor (vWF) [Contains: von Willebrand antigen 2 (von Willebrand antigen II)] - Homo sapiens (Human)	HUMAN	1
124	2.84	3.04	15.3	P33151|CADH5_HUMAN	Cadherin-5 precursor (Vascular endothelial-cadherin) (VE-cadherin) (7B4 antigen) (CD144 antigen) - Homo sapiens (Human)	HUMAN	1
125	2.81	3.87	28.2	P35908|K22E_HUMAN	Keratin, type II cytoskeletal 2 epidermal (Cytokeratin-2e) (K2e) (CK 2e) - Homo sapiens (Human)	HUMAN	1
126	2.81	3.35	8.6	Q9P2P6|STAR9_HUMAN	StAR-related lipid transfer protein 9 (StARD9) (START domain-containing protein 9) (Fragment) - Homo sapiens (Human)	HUMAN	1
127	2.74	2.79	14.8	P18428|LBP_HUMAN	Lipopolysaccharide-binding protein precursor (LBP) - Homo sapiens (Human)	HUMAN	1
128	2.71	3.1	27.8	P17936|IBP3_HUMAN	Insulin-like growth factor-binding protein 3 precursor (IGFBP-3) (IBP-3) (IGF-binding protein 3) - Homo sapiens (Human)	HUMAN	1
129	2.68	3.87	22.5	P35579|MYH9_HUMAN	Myosin-9 (Myosin heavy chain 9) (Myosin heavy chain, nonmuscle IIa) (Nonmuscle myosin heavy chain IIa) (NMMHC II-a) (NMMHC-IIA)	HUMAN	1
130	2.67	2.9	15.4	RRRRRP19174|PLCG1_HUM	REVERSED 1-phosphatidylinositol-4,5-bisphosphate phosphodiesterase gamma 1 (EC 3.1.4.11) (Phosphoinositide phospholipase C)	HUMAN	1
132	2.64	2.64	22.6	Q8N9L9|ACOT4_HUMAN	Acyl-coenzyme A thioesterase 4 (EC 3.1.2.2) (Acyl-CoA thioesterase 4) (Peroxisomal acyl coenzyme A thioester hydrolase Ib) (Peroxisom	HUMAN	1
134	2.6	2.64	15.6	Q96PD5|PGRP2_HUMAN	N-acetylmuramoyl-L-alanine amidase precursor (EC 3.5.1.28) (Peptidoglycan recognition protein long) (PGRP-L) (Peptidoglycan recogni	HUMAN	1
135	2.59	2.59	29.1	P08571|CD14_HUMAN	Monocyte differentiation antigen CD14 precursor (Myeloid cell-specific leucine-rich glycoprotein) [Contains: Monocyte differentiation a	HUMAN	1
136	2.58	2.6	12.6	P00740|FA9_HUMAN	Coagulation factor IX precursor (EC 3.4.21.22) (Christmas factor) (Plasma thromboplastin component) (PTC) [Contains: Coagulation fact	HUMAN	1
138	2.46	2.63	19	Q14764|MVP_HUMAN	Major vault protein (MVP) (Lung resistance-related protein) - Homo sapiens (Human)	HUMAN	1
139	2.44	2.47	27.9	RRRRRQ9BV73|CP250_HUM	REVERSED Centrosome-associated protein CEP250 (Centrosomal protein 2) (Centrosomal Nek2-associated protein 1) (C-Nap1) - Homo s	HUMAN	1
141	2.43	3.16	15.4	O60306|AQR_HUMAN	Intron-binding protein aquarius (Intron-binding protein of 160 kDa) (IBP160) - Homo sapiens (Human)	HUMAN	1
142	2.41	6.17	18.5	RRRRRQ8TE73|DYH5_HUMA	REVERSED Ciliary dynein heavy chain 5 (Axonemal beta dynein heavy chain 5) (HL1) - Homo sapiens (Human)	HUMAN	1
143	2.4	2.7	10.1	RRRRRO75592|MYCB2_HU	REVERSED Probable E3 ubiquitin-protein ligase MYCBP2 (EC 6.3.2.-) (Myc-binding protein 2) (Protein associated with Myc) (Pam/highwi	HUMAN	1
144	2.37	2.46	15.2	RRRRRP28715|ERCC5_HUM	REVERSED DNA-repair protein complementing XP-G cells (Xeroderma pigmentosum group G-complementing protein) (DNA excision re	HUMAN	1
145	2.36	2.36	43.6	P81605|DCD_HUMAN	Dermcidin precursor (Preproteolysin) [Contains: Survival-promoting peptide; DCD-1] - Homo sapiens (Human)	HUMAN	1
146	2.32	2.78	20.4	Q8TEW0|PARD3_HUMAN	Partitioning-defective 3 homolog (PARD-3) (PAR-3) (Atypical PKC isotype-specific-interacting protein) (ASIP) (CTCL tumor antigen se2-5)	HUMAN	1
147	2.31	3.3	11	RRRRRP46721|SO1A2_HUM	REVERSED Solute carrier organic anion transporter family member 1A2 (Solute carrier family 21 member 3) (Sodium-independent orga	HUMAN	1
148	2.3	2.33	14.5	RRRRRQ15058|KIF14_HUM	REVERSED Kinesin-like protein KIF14 - Homo sapiens (Human)	HUMAN	1
149	2.27	2.5	14.1	Q9Y490|TLN1_HUMAN	Talin-1 - Homo sapiens (Human)	HUMAN	1
150	2.26	3.35	16.3	RRRRRQ8N7A1|KLDC1_HU	REVERSED Kelch domain-containing protein 1 - Homo sapiens (Human)	HUMAN	1
153	2.2	2.38	19	Q15582|BGH3_HUMAN	Transforming growth factor-beta-induced protein ig-h3 precursor (Beta ig-h3) (Kerato-epithelin) (RGD-containing collagen-associated p	HUMAN	1
154	2.2	2.26	13.8	Q75V66|TM16E_HUMAN	Transmembrane protein 16E (Gnathodiaphyseal dysplasia 1 protein) - Homo sapiens (Human)	HUMAN	1
155	2.2	2.23	14.1	RRRRRQ08999|RBL2_HUMA	REVERSED Retinoblastoma-like protein 2 (130 kDa retinoblastoma-associated protein) (PRB2) (P130) (RBR-2) - Homo sapiens (Human)	HUMAN	1
157	2.17	4.49	25.5	O15078|CE290_HUMAN	Centrosomal protein Cep290 (Nephrocystin-6) (Tumor antigen se2-2) - Homo sapiens (Human)	HUMAN	1
158	2.16	2.19	11.3	RRRRRP53618|COPB_HUMA	REVERSED Coatomer subunit beta (Beta-coat protein) (Beta-COP) - Homo sapiens (Human)	HUMAN	1
159	2.16	2.18	13.3	RRRRRQ96JQ0|PCD16_HUM	REVERSED Protocadherin-16 precursor (Dachsous-1) (Cadherin-19) (Fibroblast cadherin 1) - Homo sapiens (Human)	HUMAN	1
160	2.15	2.37	15.8	Q8TEK3|DOT1L_HUMAN	Histone-lysine N-methyltransferase, H3 lysine-79 specific (EC 2.1.1.43) (Histone H3-K79 methyltransferase) (H3-K79-HMTase) (DOT1-like	HUMAN	1
161	2.15	2.15	16.8	P00748|FA12_HUMAN	Coagulation factor XII precursor (EC 3.4.21.38) (Hageman factor) (HAF) [Contains: Coagulation factor XIIa heavy chain; Beta-factor XIIa p	HUMAN	1
162	2.13	3.05	16.8	Q13099|IFT88_HUMAN	Intraflagellar transport 88 homolog (Tetratricopeptide repeat protein 10) (TPR repeat protein 10) (Recessive polycystic kidney disease p	HUMAN	1
163	2.13	2.2	15.7	O76024|WFS1_HUMAN	Wolframin - Homo sapiens (Human)	HUMAN	1
165	2.12	2.29	29.8	P63104|1433Z_HUMAN	14-3-3 protein zeta/delta (Protein kinase C inhibitor protein 1) (KCIP-1) - Homo sapiens (Human)	HUMAN	1
166	2.11	2.79	7.2	RRRRRP78509|RELN_HUMA	REVERSED Reelin precursor (EC 3.4.21.-) - Homo sapiens (Human)	HUMAN	1
167	2.11	2.15	20.3	P55058|PLTP_HUMAN	Phospholipid transfer protein precursor (Lipid transfer protein II) - Homo sapiens (Human)	HUMAN	1
168	2.11	2.11	16.5	O60858|TRI13_HUMAN	Tripartite motif-containing protein 13 (Ret finger protein 2) (Putative tumor suppressor RFP2) (Leukemia-associated protein 5) (B-cell c	HUMAN	1
169	2.11	2.11	25.7	P61626|LYSC_HUMAN	Lysozyme C precursor (EC 3.2.1.17) (1,4-beta-N-acetylmuramidase C) - Homo sapiens (Human)	HUMAN	1
170	2.11	2.11	17.5	P08294|SODE_HUMAN	Extracellular superoxide dismutase [Cu-Zn] precursor (EC 1.15.1.1) (EC-SOD) - Homo sapiens (Human)	HUMAN	1
171	2.1	2.92	11.2	Q9HCF4|ALO17_HUMAN	Protein ALO17 (ALK lymphoma oligomerization partner on chromosome 17) - Homo sapiens (Human)	HUMAN	1
172	2.1	2.27	8.6	RRRRRQ9H422|HIPK3_HUM	REVERSED Homeodomain-interacting protein kinase 3 (EC 2.7.11.1) (Homolog of protein kinase YAK1) (Fas-interacting serine/threonine	HUMAN	1
173	2.09	2.09	15.3	O75167|PHAR2_HUMAN	Phosphatase and actin regulator 2 - Homo sapiens (Human)	HUMAN	1
174	2.08	2.22	15.4	Q6WCQ1|MRIP_HUMAN	Myosin phosphatase Rho-interacting protein (Rho-interacting protein 3) (M-RIP) (RIP3) (p116Rip) - Homo sapiens (Human)	HUMAN	1
175	2.08	2.08	30.5	P02775|SCYB7_HUMAN	Platelet basic protein precursor (PBP) (Small inducible cytokine B7) (CXCL7) (Leukocyte-derived growth factor) (LDGF) (Macrophage-der	HUMAN	1
177	2.07	2.33	10.8	Q9UGJ1|GCP4_HUMAN	Gamma-tubulin complex component 4 (GCP-4) (hGCP4) (h76p) (Hgrip76) - Homo sapiens (Human)	HUMAN	1
178	2.06	2.15	13.4	RRRRRQ6P0Q8|MAST2_HU	REVERSED Microtubule-associated serine/threonine-protein kinase 2 (EC 2.7.11.1) - Homo sapiens (Human)	HUMAN	1
179	2.05	2.61	16.4	Q96GE4|CCD45_HUMAN	Coiled-coil domain-containing protein 45 - Homo sapiens (Human)	HUMAN	1
180	2.05	2.05	20.9	Q15848|ADIPO_HUMAN	Adiponectin precursor (Adipocyte, C1q and collagen domain-containing protein) (30 kDa adipocyte complement-related protein) (Adipo	HUMAN	1
182	2.04	2.08	11.4	RRRRRQ9UQQ1|NALDL_HU	REVERSED N-acetylated-alpha-linked acidic dipeptidase-like protein (EC 3.4.17.21) (NAALADase L) (Ileal dipeptidylpeptidase) (100 kDa il	HUMAN	1
183	2.03	2.68	26.4	Q7Z406|MYH14_HUMAN	Myosin-14 (Myosin heavy chain 14) (Myosin heavy chain, nonmuscle IIc) (Nonmuscle myosin heavy chain IIc) (NMHC II-C) - Homo sapie	HUMAN	1
184	2.03	2.13	20.8	RRRRRQ9UNZ2|NSF1C_HU	REVERSED NSFL1 cofactor p47 (p97 cofactor p47) - Homo sapiens (Human)	HUMAN	1
185	2.03	2.11	20.5	RRRRRO14776|TCRG1_HUM	REVERSED Transcription elongation regulator 1 (TATA box-binding protein-associated factor 2S) (Transcription factor CA150) - Homo sa	HUMAN	1
186	2.03	2.03	19.5	Q9UJH8|METRN_HUMAN	Meteorin precursor - Homo sapiens (Human)	HUMAN	1
187	2.02	2.8	18	Q8IY37|DHX37_HUMAN	Probable ATP-dependent RNA helicase DHX37 (EC 3.6.1.-) (DEAH box protein 37) - Homo sapiens (Human)	HUMAN	1
188	2.02	2.2	13.4	RRRRRO60885|BRD4_HUM	REVERSED Bromodomain-containing protein 4 (HUNK1 protein) - Homo sapiens (Human)	HUMAN	1
189	2.02	2.18	11.1	Q02763|TIE2_HUMAN	Angiopoietin-1 receptor precursor (EC 2.7.10.1) (Tyrosine-protein kinase receptor TIE-2) (hTIE2) (Tyrosine-protein kinase receptor TEK)	HUMAN	1
190	2.02	2.11	10.5	RRRRRQ6PKC3|TXD11_HUM	REVERSED Thioredoxin domain-containing protein 11 (EF-hand-binding protein 1) - Homo sapiens (Human)	HUMAN	1
191	2.02	2.1	16.5	RRRRRO60347|TBC12_HUM	REVERSED TBC1 domain family member 12 - Homo sapiens (Human)	HUMAN	1
192	2.02	2.07	14.8	RRRRRQ9NTZ6|RBM12_HU	REVERSED RNA-binding protein 12 (RNA-binding motif protein 12) (SH3/WW domain anchor protein in the nucleus) (SWAN) - Homo	HUMAN	1
193	2.02	2.07	6.8	Q9Y6C5|PTC2_HUMAN	Protein patched homolog 2 (PTC2) - Homo sapiens (Human)	HUMAN	1
194	2.02	2.05	15.6	RRRRRP0C025|NUD17_HUM	REVERSED Putative nucleoside diphosphate-linked moiety X motif 17, mitochondrial precursor (EC 3.6.1.-) (Nudix motif 17) - Homo sapi	HUMAN	1
197	2.01	2.06	7.5	Q00610|CLH1_HUMAN	Clathrin heavy chain 1 (CLH-17) - Homo sapiens (Human)	HUMAN	1
198	2.01	2.04	19.2	RRRRRQ86TV6|TTC7B_HUM	REVERSED Tetratricopeptide repeat protein 7B (TPR repeat protein 7B) (Tetratricopeptide repeat protein 7-like-1) - Homo sapiens (Hum	HUMAN	1
199	2.01	2.01	17.2	RRRRRQ8IVH4|MMAA_HU	REVERSED Methylmalonic aciduria type A protein, mitochondrial precursor - Homo sapiens (Human)	HUMAN	1
200	2.01	2.01	41	P02735|SAA_HUMAN	Serum amyloid A protein precursor (SAA) [Contains: Amyloid protein A (Amyloid fibril protein AA); Serum amyloid protein A(2-104); Ser	HUMAN	1
201	2	3.01	12.5	P10720|PF4V_HUMAN	Platelet factor 4 variant precursor (PF4var1) (PF4alt) (CXCL4L1) [Contains: Platelet factor 4 variant(4-74); Platelet factor 4 variant(5-74);	HUMAN	1
202	2	2.15	11.5	O00187|MASP2_HUMAN	Mannan-binding lectin serine protease 2 precursor (EC 3.4.21.104) (Mannose-binding protein-associated serine protease 2) (MASP-2) (	HUMAN	1
203	2	2.14	15.8	P07477|TRY1_HUMAN	Trypsin-1 precursor (EC 3.4.21.4) (Trypsin I) (Cationic trypsinogen) (Serine protease 1) - Homo sapiens (Human)	HUMAN	1
204	2	2.07	13.3	P11226|MBL2_HUMAN	Mannose-binding protein C precursor (MBP-C) (MBP1) (Mannan-binding protein) (Mannose-binding lectin) - Homo sapiens (Human)	HUMAN	1
205	2	2.06	15.8	RRRRRQ8TBB0|THAP6_HUM	REVERSED THAP domain-containing protein 6 - Homo sapiens (Human)	HUMAN	1
206	2	2.04	14.6	RRRRRQ02763|TIE2_HUMA	REVERSED Angiopoietin-1 receptor precursor (EC 2.7.10.1) (Tyrosine-protein kinase receptor TIE-2) (hTIE2) (Tyrosine-protein kinase rec	HUMAN	1
207	2	2.04	18.1	Q9UGM5|FETUB_HUMAN	Fetuin-B precursor (Gugu) (IRL685) (16G2) - Homo sapiens (Human)	HUMAN	1
208	2	2.02	36.1	P61769|B2MG_HUMAN	Beta-2-microglobulin precursor [Contains: Beta-2-microglobulin variant pI 5.3] - Homo sapiens (Human)	HUMAN	1

209	2	2.01	9.4	P16070|CD44_HUMAN	CD44 antigen precursor (Phagocytic glycoprotein I) (PGP-1) (HUTCH-I) (Extracellular matrix receptor-III) (ECMR-III) (GP90 lymphocyte h	HUMAN	1
210	2	2.01	21.9	P01591|IGJ_HUMAN	Immunoglobulin J chain - Homo sapiens (Human)	HUMAN	1
211	2	2	8.9	RRRRRO75928|PIAS2_HUM	REVERSED E3 SUMO-protein ligase PIAS2 (Protein inhibitor of activated STAT2) (Protein inhibitor of activated STAT x) (Msx-interacting z	HUMAN	1
212	2	2	14	Q9Y5Y7|XLKD1_HUMAN	Lymphatic vessel endothelial hyaluronic acid receptor 1 precursor (LYVE-1) (Cell surface retention sequence-binding protein 1) (CRSBP-	HUMAN	1
213	2	2	17.5	P00441|SODC_HUMAN	Superoxide dismutase [Cu-Zn] (EC 1.15.1.1) - Homo sapiens (Human)	HUMAN	1
214	2	2	26.7	P31151|S10A7_HUMAN	Protein S100-A7 (S100 calcium-binding protein A7) (Psoriasin) - Homo sapiens (Human)	HUMAN	1

Supplementary table 2

Protein	1st set (113:117)	p value	2nd set (114:118)	p value	3rd set (115:119)	p value	4th set (114:116)	p value	5th set (115:117)	p value	technical rep_(116: 121)	p value
Actin, Cytoplasmic 2	1.6169	0.0043	0.9357	0.7352	1.4223	0.3698	1.0331	0.8705	2.4749	0.0002	1.5159	0.006
Adiponectin	0.683		1.2263		0.9485		0.7327	0.0667	0.5968	0.1077	0.5345	
Alpha-1-acid glycoprotein 2	0.7813	0.0864	0.9165	0.5213	0.5037	0.3088	0.948	0.9518	0.6942	0.3549	0.8198	0.1094
AMBP protein	1.1621	0.3048	1.3553	0.0071	1.6125	0.157	1.532	0.0118	0.7091	0.1445	1.1644	0.2282
Apolipoprotein A-I	0.6278	0.0328	1.0048	0.9769	0.3461	0.0959	0.457	0.3821	0.9389	0.848	0.7574	0.0483
Apolipoprotein A-IV	0.7265	0	0.8253	0.0001	0.6901	0.0189	1.001	0.9967	0.7471	0	0.7997	0
Apolipoprotein B-100	0.7909	0	0.8079	0.0003	0.7438	0.0139	0.787	0.0123	0.8872	0.0024	0.84	0
Apolipoprotein C-I	0.7838	0.0161	0.486	0.2224	0.4974	0.0531	0.2886	0.0057	0.9913	0.9579	0.8224	0.0258
Apolipoprotein C-II	1.5611	0.201	0.73	0.4816	0.5236	0.0289	0.3934	0.0658	1.2617	0.02	1.4146	0.2134
Apolipoprotein L1	0.9343	0.4138	0.7858	0.1247	1.1346	0.586	0.6109	0.0484	0.7831	0.0116	0.8622	0.0528
Dermcidin	1.7836	0.2408	1.4488	0.1714	4.6536	0.4231	1.1599	0.6884	2.0635	0.3698	1.2951	0.1859
Fibrinogen gamma chain	0.7686	0.0106	1.1267	0.571	1.4741	0.4671	1.4227	0.2892	1.2637	0.1382	0.9152	0.2147
Glutathione peroxidase 3	0.7084	0.0756	1.2212	0.2618	0.4706	0.0227	0.7414	0.6258	1.0727	0.7439	0.8432	0.1652
Peroxiredoxin-2	1.3775	0.1879	0.8013	0.2086	2.1358	0.5221	0.9135		1.2761		0.9777	0.9337
Pregnancy zone protein	1.2713	0.2163	0.9843	0.8503	1.2544	0.4463	1.9943	0.106	1.5681	0.1053	0.7533	0.3402
Serum amyloid P-component	0.6806	0.0004	1.0889	0.4786	0.3114	0.0018	0.3759	0.0444	0.7074	0.0705	0.706	0.0119
Thymosin beta-4	2.2765		1.5369		0.8007		2.4334		4.0036		1.6515	
Vitronectin	0.9495	0.6464	1.4318	0.0157	1.0016	0.9974	1.2879	0.0353	1.363	0.0008	1.1746	0.0005

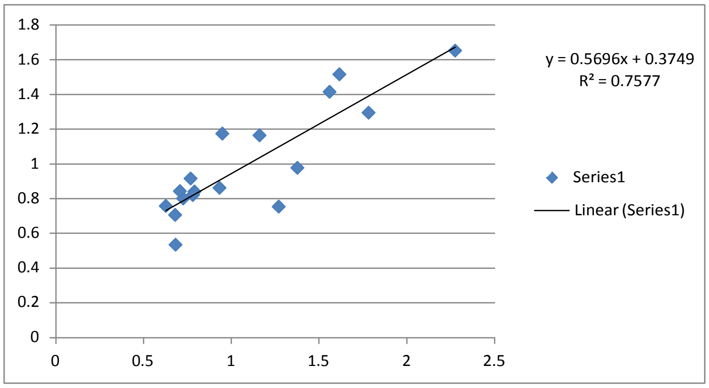

Protein	CAD(NOT ON STATIN) (N=147)	CONTROL (N=253)	P-VALUE
APO AIV (ng/ml)	33979.96(27327.40-43273.72)	41168.18(33699.40-51181.45)	8.13E-07
APOC1(µg/ml)	121.07(87.04-163.33)	144.99(116.25-181.75)	9.49E-06
ALB (g/dL)	3.8(3.6-4.1)	4.3(4.1-4.5)	3.62E-26
APOAI (mg/dL)	93.54(78.56-104.17)	114.38(101.14-130.31)	9.63E-25

Mann-whitney test

Supplementary table 4

Protein	Hypertensive Control (N=38)	Non-hypertensive Control (N=215)	p-VALUE
ALBUMIN (g/dl)	4.4(4.05-4.6)	4.3(4.1-4.5)	0.56
Protein	Hypertensive CAD (N=149)	Non-Hypertensive CAD (N=104)	p-VALUE
ALBUMIN (g/dl)	3.7(3.5-4.07)	3.8(3.5-4.1)	0.25
